# Sequence-robust MRI asymmetry measures for the detection of anoperineal lesions in Crohn’s disease on axial pelvic MRI scans

**DOI:** 10.1371/journal.pone.0340243

**Published:** 2026-07-21

**Authors:** Corentin Lucas, Myriam Bontonou, Marc Aubry, Eric Ogier-Denis, Guillaume Bouguen, Emmanuelle Becker, Yann Le Cunff

**Affiliations:** 1 Equipe BioGraphs, Univ Rennes, Inria, CNRS, IRISA - UMR, Rennes, France; 2 Univ Rennes, INSERM, OSS (Oncogenesis Stress Signaling), UMR_S, CLCC Eugene Marquis, F-35000, Rennes, France; 3 CHU Rennes, Univ Rennes, INSERM CIC1414, Institut NUMECAN (Nutrition Metabolism and Cancer) F-35000, Rennes, France; Islamia University of Bahawalpur: The Islamia University of Bahawalpur Pakistan, PAKISTAN

## Abstract

Magnetic resonance imaging (MRI) is widely considered the gold standard for evaluating Crohn’s disease anoperineal lesions. However, despite its central role in clinical evaluation, automated methods specifically dedicated to the analysis of these lesions remain rare. This is particularly challenging in the context of anoperineal lesions due to (i) the diversity of MRI sequences, (ii) the small size of cohorts available to train complex models, and (iii) the absence of a standard shape for lesions. In this context, we explore an alternative strategy based on interpretable asymmetry features in axial MRIs, explicitly capturing morphological and intensity differences between the left and right sides of the perineal region. These features were used to either classify images as containing or not anoperineal lesions, or to precisely locate a lesion on an MRI slice. We apply this method on a small cohort of 44 patients affected by Crohn’s disease, including 134 sequences of MRI of various types (38 T1 sequences, 74 T2 sequences, etc). First, a proof-of-concept dataset was constructed, including images either clearly presenting lesions or not (thus excluding symmetrical lesions), to prove that the asymmetry features we compute are sensitive to the presence of asymmetric lesions on MRI images. Importantly, we were able to show that the asymmetry features are not particularly sensitive to MRI sequences’ type, or patients’ effect, even with limited annotated datasets. The classification model demonstrates promising diagnostic performance, achieving an Area Under Curve (AUC) greater than 0.85 on the proof-of-concept dataset. However, when making predictions on the validation and test dataset, the AUCs drop to 0.667 and 0.647 respectively. The model struggles to generalize to all kinds of lesions, particularly to classify lesions slices when symmetrical (horseshoe) lesions are observed. Additionally, the method designed to locate a lesion in an image exhibited good precision, with a median distance of 1 centimeter between the detected area and the actual lesion position on our proof-of-concept dataset and 1.2 centimeter on the complete dataset, indicating its potential usefulness in future studies focused on standardized assessment of anoperineal lesions.

## Introduction

Crohn’s disease is classified as an Inflammatory Bowel Disease (IBD) – more precisely, a chronic inflammatory bowel condition – that significantly affects patients’ quality of life [1,2]. Various complications can occur in patients, among which anoperineal lesions represent about 30% of cases [3] and constitute a clinical challenge [4,5]. Magnetic Resonance Imaging (MRI) is currently the standard method for detecting anoperineal lesions, whose early identification is critical for appropriate management and treatment [6–8]. These lesions correspond to tissue anomalies, a type of pattern that deep learning methods have successfully detected in other MRI studies owing to (i) the large number of available images for model training [9–13], (ii) standardized imaging protocols [9,11,14], and (iii) well-characterized anomalies [13,14]. However, these conditions are generally not met in anoperineal Crohn’s disease MRI cohorts.

Deep learning approaches applied to MRI typically rely on several hundred independent patients, producing thousands of MRI sequences and tens of thousands of images. For example, MADGAN, developed by Han et al., is a Generative Adversarial Network for detecting anomalies in brain MRI slices which was trained on the public OASIS-3 dataset, using several hundred patients and thousands of MRI sequences [15]. Similarly, Saeedi et al. designed a Convolutional Neural Network for brain tumor classification based on more than three thousand T1-weighted contrast-enhanced MRI images [16]. After preprocessing to a standardized resolution, data augmentation through vertical and horizontal rotations increased the dataset to about ten thousand images. By contrast, anoperineal Crohn’s disease MRI cohorts remain limited, usually comprising 25–50 patients [8,17–19].

Beyond dataset size, deep learning approaches applied to MRI rely on relatively homogeneous image content and standardized-resolution MRI sequences. For example, despite four tumor classes, the images in [16] share similar visual characteristics, typically appearing as round masses within the darker brain outline. More generally, deep learning studies focus on a single MRI modality and adapt models specifically to that type [13,15]. Deep learning methods also depend on controlled acquisition protocols, consistent data formats, and well-defined anomaly types to ensure reproducible performance [20,21]. Anoperineal Crohn’s disease imaging does not meet these criteria. There is no standardized MRI protocol for this condition: T1, T2, contrast-enhanced, or other sequences can be used to identify lesions, resulting in heterogeneous datasets [22]. In addition, anoperineal lesions can be classified as complex or simple. Complex lesions vary across MRI slices in shape, number of tracts, and position across planes [23], making prior characterization difficult. Such heterogeneity limits the ability of deep learning models to generalize and learn consistent features across patients.

Consequently, few studies have applied deep learning to MRI analysis of Crohn’s disease. Existing work has focused on identifying Crohn’s tissue in Magnetic Resonance Enterography [24] or distinguishing Crohn’s disease from Ulcerative Colitis using endoscopic images from public datasets (LIMUC, HyperKvasir, and CrohnIPI) [25]. To date, no studies have addressed the automatic detection of anoperineal lesions on Crohn’s disease MRI. Given the lack of large, transferable deep learning models suitable for this setting, MRI studies of anoperineal lesions in Crohn’s disease rely on manual annotation and interpretation by expert radiologists.

Outside deep-learning approaches, computational methods can however propose complementary approaches that are simpler but easily interpretable. Among these, robust techniques such as asymmetry-based analyses have been used in other imaging contexts such as brain [14] or breast MRI [26], and may provide relevant methodological candidates for application to anoperineal lesion detection.

Asymmetry-based approaches assume that certain anatomical structures are naturally symmetrical and that deviations from this symmetry indicate potential abnormalities. They do not require predefined shapes for detection. Quantitative measures such as the Mean Absolute Error (MAE) and the Structural Similarity Index Measure (SSIM) are suitable for image comparison and anomaly detection [27]. These techniques are computationally less complex and more interpretable than deep learning models, making them suitable for small or heterogeneous cohorts.

In this study, we evaluate asymmetry measures as potential biomarkers for detecting and localizing anoperineal lesions on axial pelvic MRI. We first explore various asymmetry measures to identify those sensible to anoperineal lesion presence/absence, independently of MRI type. We then explore the potential of the selected asymmetry measures to detect anoperineal lesions, or to localize precisely anoperineal lesions, independently of MRI type.

## Materials and methods

### Description of the cohort and data

**Fig 1:**
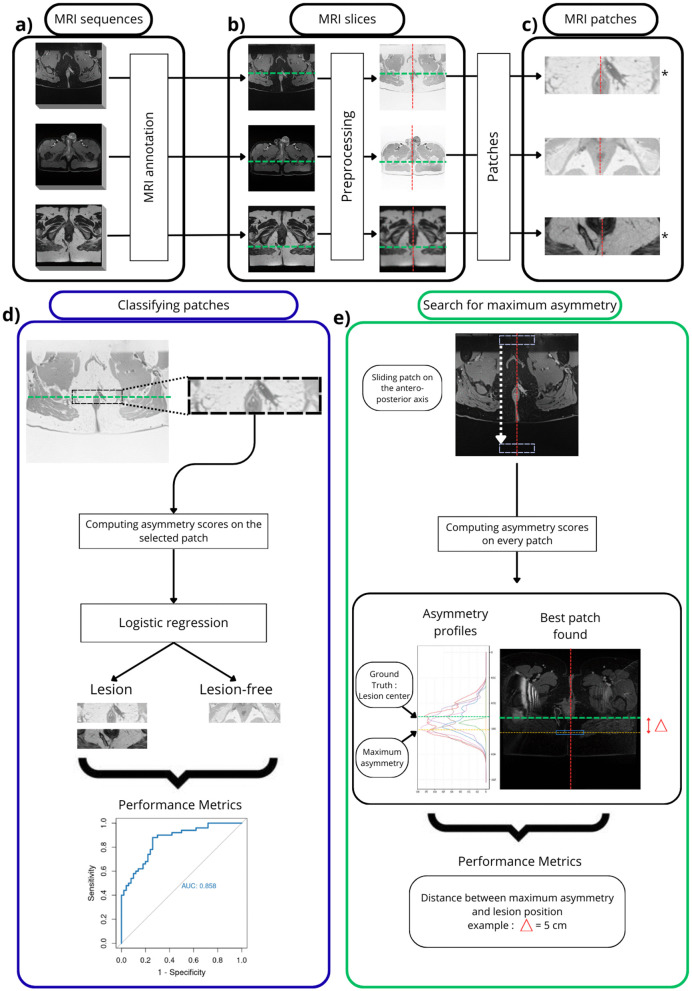
Pipeline overview. a) The dataset contains a variety of MRI sequences with different zoom levels, contrasts, and resolutions. For each sequence, an annotation is provided to identify slices with lesions in an MRI sequence and the lesion position on slices (green dotted line). b) Each MRI sequence is composed of MRI slices, which undergo several preprocessing steps. We first use a gaussian filter before computing the best symmetry axis (red dotted line) and finally we apply discrete wavelet transform. c) Each preprocessed slice has a patch sampled from it. Depending on the slice from which it was sampled, this patch may or may not contain a lesion (lesion patches illustrated with an “*”). d) To determine whether an asymmetry exists in a patch, a logistic regression model is trained to classify patches based on asymmetry scores. e) On a slice, patches are extracted by sliding a window along the symmetry axis, producing an asymmetry profile for each asymmetry score. From this profile we identify the most likely region to contain the lesion on the MRI slice (see section Asymmetry scores enable locating lesions on a single MRI slice).

The data were extracted from the 3TLAP cohort, a French cohort from the Hospital-University Center of Rennes (CHU Rennes) that includes patients affected by Crohn’s disease with ano-perineal lesions. This cohort was approved by the “Comité de Protection des Personnes SUD-EST IV” ethics committee. All participants provided written informed consent and were prospectively included starting July 9, 2019, and ending January 5, 2025. We initially obtained the data on January 3, 2024, and all MRI data were anonymized. The cohort includes 52 patients with axial MRIs, composed of 20–256 slices, available in DICOM format. Of these 52 patients, 3 were excluded because no lesion was detected on MRI due to previous surgery, and 5 others were excluded because of low MRI quality, resulting in a cohort of 44 patients available for this study (Table 1). Each patient may have several types of MRI available in the dataset, such as T1, T2, or LAVA-FLEX (Fig 1).

**Table 1:** Summary of MRI sequences in the dataset.

Variable	Value
Total number of patients	**44**
Total number of MRI sequences	**134**
Mean number of MRI sequences per patient	**3.15 (min: 1, max: 8)**
Minimum number of slices per sequence	**20**
Maximum number of slices per sequence	**256**
Total number of T1 sequences	**38**
Average number of slices per T1	**83.0 (min: 20, max: 256)**
Total number of T2 sequences	**74**
Average number of slices per T2	**27.7 (min: 20, max: 36)**
Total number of LAVA-FLEX sequences	**16**
Average number of slices per LAVA-FLEX	**193.2 (min: 100, max: 232)**
Total number of other sequences	**6**
Average number of slices per other sequence	**30.7 (min: 24, max: 56)**

For each MRI sequence, each slice was manually annotated as presenting or not presenting a lesion, thus defining a continuous series of slices in which the lesion is visible, called the “lesion range.” The central slice of the lesion range was considered representative to assign a precise location (X, Y) to the lesion across the entire MRI sequence.

### Overview of the workflow

As presented in Fig 1, the workflow begins with the annotation of all MRI sequences (Fig 1, a) and the pre-processing of MRI slices (Fig 1, b). Each slice is processed to extract rectangular patches centered on the global symmetry axis (Fig 1, c), and ultimately asymmetry measures are computed on each patch.

Two workflows were designed to perform two tasks, for which the patch selection differs:

1. The first task (Fig 1, d) is to classify the patches as containing a lesion or not, using asymmetry measures as input features for a logistic regression model. For this task, all patches are placed at the exact height of the lesion, in slices inside or outside the lesion range.
2. The second task is to automatically identify the location of a lesion within a slice containing one (Fig 1, e). For this task, patches are extracted using a sliding window along the symmetry axis of the slice (sliding window with a step size of one pixel). The lesion is predicted to be the center of the patch with the maximal asymmetry, based on a global asymmetry score that we call the 3-Peak score. (see section “Task 2: Positioning a lesion on a slice”)

Each step of the workflows is further detailed in the following sections. The code is available through the following link: https://github.com/CoLucas22/MRI_Asymmetry_Analysis_Pipeline.

### Preprocessing MRI slices

Each slice is subjected to a series of pre-processing steps. First, a Gaussian filter is applied to reduce noise impact on the MRI image. Next, the bilateral symmetry axis of the body is calculated, as the body is not always perfectly centered in the MRI. Finally, a discrete wavelet transform is applied to obtain an alternative representation of the slice.

### Noise impact reduction

We use a Gaussian filter to reduce the noise present in the MRI sequences. First, it is applied to compute a symmetry axis and to find a global symmetry axis without taking every small detail into account. Second, it is used before computing the asymmetry scores to reduce the impact of noise. The Gaussian filter provided by the SciPy Python package was used. The Gaussian kernel has a radius of 5 and a standard deviation of 1.

### Symmetry axis positioning

The objective is to find an overall symmetry axis that allows the image to be viewed as a whole, without too much detail, in order to detect the symmetry of the human body in the anorectal region.

To obtain the best symmetry axis on each slice, we try different positions and angles of this axis and search for the best correlation score between the two halves of the slice.

To maximize the precision of the detected symmetry axis, we divide the slice into *k* horizontal strips and compute an independent symmetry axis per strip, resulting in *k* symmetry axis. To compute one symmetry axis, we assume that DICOM files are approximately horizontally centered, and start by placing the axis in the middle of the strip. We then test different combinations of offset and angle, allowing an offset of ±10% of the pixel width of the slice and an angle of ±5 degrees, and select the one with the highest correlation value between the left and right parts of the axis using the corrcoef function from the NumPy package.


$$\begin{array}{c} r=\frac{\sum_{i=1}^{N}(X_{i}-\mu_{X})(Y_{i}-\mu_{Y})}{\sqrt{\sum_{i=1}^{N}(X_{i}-\mu_{X}{)}^{2}}\;\sqrt{\sum_{i=1}^{N}(Y_{i}-\mu_{Y}{)}^{2}}} \end{array}$$ (1)

where *X* and *Y* are the left and right halves of the slice, each containing *N* pixels; $X_{i}$ and $Y_{i}$ are the pixel intensity values of the *i*-th pixel, with $X_{i},Y_{i}\in \{0,1,\ldots ,255\}$; and $\mu_{X}$ and $\mu_{Y}$ are the mean pixel intensities of *X* and *Y*, respectively.

Once the positions of the *k* symmetry axes are defined, we compute the median symmetry axis. Using the median instead of the mean symmetry axis helps remove the effect of the lesion on the axis location. If the lesion is large and bright in an otherwise dark MRI, the correlation formula will be biased and tend to locate the symmetry axis in the middle of the lesion.

### Discrete Wavelet transform

We used the Python package PyWavelets. After applying a Gaussian filter to reduce noise, we performed a discrete wavelet transform using the Haar wavelet. As a result, we used the horizontal, vertical, and diagonal coefficients obtained from this transform.

### Description of the datasets

The study cohort comprised 44 patients, represented by 134 MRI sequences. For each sequence, four slices were extracted: two slices centered on the lesion area (hereinafter “lesion slices”) and two slices located outside this area but within the perineal region (when available), serving as controls. All patients were divided into two subgroups: a group of 33 patients dedicated to method development and the selection of asymmetry scores, and a group of 11 patients reserved for the final evaluation using completely independent data.

#### 33 Patients.

The 33 patients in the development group are represented by 95 MRI sequences, comprising a total of 364 extracted slices, from which two distinct subsets were created:

- **Proof of Concept (PoC).** This subset aims to verify the hypothesis that asymmetry is a relevant indicator for the detection of anoperineal lesions. It comprises 100 MRI slices (50 with lesions / 50 lesion-free), selected based on criteria of visual representativeness: clear visibility of the lesion for pathological slices, and marked symmetry for healthy slices. As a result, this subset does not reflect the full diversity of MRI scans in the cohort. It was used to identify significant asymmetry measures and to train a logistic regression model.
- **Validation dataset.** This subset comprises the remaining 264 slices, drawn from the same patient cohort as the PoC. It allows for the evaluation of the generalization ability of the model trained on the PoC to real-world data, while examining the potential influence of the source patient on the calculated asymmetry scores.

#### 11 patients (Test dataset).

These 11 patients not included in the development phase, constitute our independent test subset. This subset comprises 39 MRI sequences and 155 slices in total. Since none of these images were used during the development of the method, this test set ensures an unbiased evaluation of the model’s final performance.

### Task 1: Classifying patches based on asymmetry scores

A classifier was trained to differentiate patches containing lesions from lesion-free patches. To this end, patches vertically centered on the lesion and horizontally centered on with the bilateral symmetry axis were extracted from the preprocessed slices (*height* = 1 cm, *width* = 7 cms). The patch dimensions were determined by examining the distribution of lesion distances relative to the MRI’s axis of symmetry, resulting in patches 7 cm wide and 1 cm high. Next, for each MRI scan, the metadata corresponding to the inter-pixel spacing (in millimeters) is retrieved to perform the cm-to-pixel conversion: the number of pixels is computed by multiplying the size in centimeters by 10 and dividing by the pixel spacing in millimeters and and then round up the result (using the GetSpacing() function from the SimpleITK Python package). The model was trained on patches from the PoC dataset. For every patch, seven asymmetry measures were computed and further used as input features. This model was then used to make predictions on the validation dataset to assess whether the limited training on the PoC dataset was sufficient to achieve good results on the validation data. Finally, the model was evaluated on the test set to assess its performance on unseen patients.

Performance metrics such as accuracy, precision, recall, and area under the ROC curve were computed to evaluate the discriminative ability of the classifier.

We used the logistic regression implementation available in the R stats library.

### Task 2: Positioning a lesion on a slice

An asymmetry score was used to determine the vertical position of a lesion on a slice containing one. On a pre-processed slice containing a lesion, a sliding window is used to scan patches vertically along the symmetry axis (*height* = 1 cm, *width* = 7 cms, *sliding window stepsize* = 1 pixel).

For each patch, the 7 asymmetry measures were computed and normalized (quantile-quantile normalization taking the non-lesion patches of the PoC dataset as the background distribution). Then, for each patch, we computed the 3-Peak Score as the average of the 3 highest normalized scores. On a given slice, once the 3-Peak Score has been computed for all patches scanned by the sliding window, the position of the lesion was predicted to be the vertical center of the patch with the highest 3-Peak Score.

### Statistical analysis

All statistical analyses were performed using R version 4.4.3. Group comparisons between two groups were conducted using the Mann–Whitney U test, with Bonferroni correction applied for multiple testing. After verifying the normality assumption, the appropriate test was selected based on homoscedasticity: Welch’s ANOVA was applied when variances were unequal, while a one-way ANOVA was used when the homoscedasticity assumption was met. Principal component analysis (PCA) was performed to reduce data dimensionality, and hierarchical ascending clustering based on Euclidean distance and the Ward.D2 linkage method was used to identify similarity patterns among patch-associated measures.

## Results

### Asymmetry scores are sensitive to lesion presence or absence

Our study relies on the assumption that most ano-perineal lesions are characterized by asymmetries visible on patient MRIs. We expect this assumption to be violated for some horseshoe lesions, but overall, we expect asymmetry to capture most cases and offer additional advantages, such as being independent of MRI types.

To begin our study, we first evaluated our assumption that asymmetry characterizes slices with lesions. We manually selected 100 slices from the 33 patients, clearly showing either ano-perineal lesions (*n* = 50) or no lesion (*n* = 50). The first step was to detect the bilateral symmetry axis on each slice. Then, we focused our analysis on a patch (height: 1 cm, width: 7 cm) containing the lesion (for lesion slices) or at the same height as the lesion (for non-lesion slices). We then computed three asymmetry scores: Jensen–Shannon divergence (JSD), Mean Absolute Error (MAE), and Structural Dissimilarity (DSSIM). Each slice was smoothed using a Gaussian filter. Scores were computed both on smoothed slices and on wavelet-transformed preprocessed slices with horizontal (cH), vertical (cV), and diagonal (cD) coefficients. Overall, this resulted in a total of twelve asymmetry values per patch.

**Fig 2:**
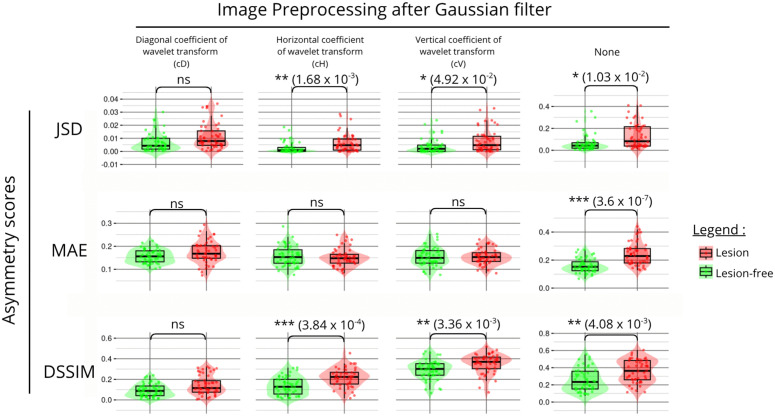
Asymmetry score distributions on the Proof-of-Concept dataset. The dataset contains 50 lesion patches and 50 lesion-free patches. For each preprocessing combination (discrete wavelet transform and Gaussian filter) applied to the MRI slices, we computed three different asymmetry scores: Jensen–Shannon Divergence (JSD), Mean Absolute Error (MAE), and Structural Dissimilarity (DSSIM). These scores were computed on the patch at the lesion position (see Fig 1.e). The distributions of lesion and non-lesion slices are displayed, and a Mann–Whitney test was performed to compare the distributions, with Bonferroni correction applied. The adjusted p-value is displayed for each significantly different distribution. The significance levels of the tests are indicated with stars: ns = non-significant, * = 0.05, ** = 0.01, *** = 0.001.

Fig 2 shows the distribution of the 12 asymmetry measures, organized in three rows (each corresponding to a base score: DSSIM, MAE, JSD) and four columns (each corresponding to a representation of the slice: Gaussian filter, Gaussian filter + cH, Gaussian filter + cV, Gaussian filter + cD). Among the twelve asymmetry measures, seven were found to be significantly sensitive to the presence or absence of lesions (Fig 2). For raw slices without preprocessing, all three asymmetry scores were significantly higher for lesion patches (adjusted p-values for DSSIM, MAE, and JSD were respectively $4.08\times 10^{-3}$, $3.6\times 10^{-7}$, and $1.03\times 10^{-2}$). For preprocessed slices, we identified four asymmetry values significantly higher in lesion patches (p-values for cH_DSSIM, cV_DSSIM, cH_JSD, and cV_JSD were respectively $3.84\times 10^{-4}$, $3.36\times 10^{-3}$, $1.68\times 10^{-3}$, and $4.92\times 10^{-2}$). We note that the wavelet transform is not efficient when applied before computing MAE-based asymmetry scores, whereas the same score performs well on Gaussian-filtered slices. For the two other scores, DSSIM and JSD, wavelet transform preprocessing improves discrimination between patches containing lesions or not, except for cD preprocessing.

**Fig 3:**
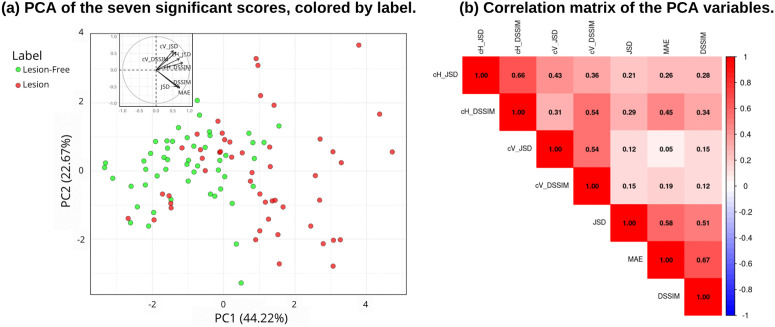
Exploratory analysis of the Proof-of-Concept dataset scores. (a) Patches are represented by their seven scores in the two main components of the PCA and colored by their label (green for patches without a lesion and red for patches with a lesion). The majority of the variance in the data (66.89%) is contained in the first two principal components. (b) Correlation matrix of the seven significant scores. The stronger the color (blue or red), the stronger Pearson correlation coefficient. The color indicates whether the data are positively (red) or negatively (blue) correlated.

This analysis validates that seven asymmetry scores are sensitive to the presence or absence of lesions in patches. To explore the combined potential of these seven scores for discriminating patches with or without lesions, we performed a Principal Component Analysis (PCA) on the seven scores and projected the label information on the principal plane (Fig 3a). We observed that patches labeled “Lesion” and “Lesion-free” were separated along the first principal component, with a Mann–Whitney test p-value of $1.9\times 10^{-8}$.

We then computed the correlation matrix of the seven significant scores (Fig 3b). We observed that the base scores were more correlated with each other than with the scores obtained after the wavelet transform, and the reverse was also true for the wavelet-derived scores. Moreover, all scores were positively correlated. The highest correlation was between MAE and DSSIM (67%), while the lowest was between JSD and MAE (0.05%), indicating that these two scores are approximately orthogonal and may capture different information about the slices. There was a lower correlation between vertical results of the wavelet transform and the base scores than between the base scores and the horizontal results of the wavelet transform.

In summary, Fig 3 suggest that all scores are consistent in detecting lesions (higher scores being associated with the presence of lesions, as shown in Fig 2) and are not completely redundant.

Additionally, for all seven significant asymmetry measures, we computed the *R*^2^ and the slope of the regression line describing the relationship between the asymmetry scores and the distance between lesion-free slices and the nearest lesion slice in the dataset. The hypothesis we aimed to test was that there is no meaningful relationship between slice distance and the potential residual asymmetry induced by the presence of a lesion on other slices. As shown in the corresponding figure, all evaluated asymmetry measures exhibit near-zero slopes and very low coefficients of determination (*R*^2^ < 0.025), indicating no meaningful relationship between distance and asymmetry.

### Asymmetry scores do not appear to be influenced MRI sequence type or patient effect

After validating the scores for lesion detection, we assessed two potential sources of bias: dependence of the scores on the type of MRI sequence and patient effect. Indeed, our dataset comprises various types of MRI data (see Material & Methods Section and Table 1), in which lesions can appear very different from one MRI type to another (e.g., high pixel intensity in T2 FatSat Propeller vs. low pixel intensity in T2 FRFSE).

**Fig 4:**
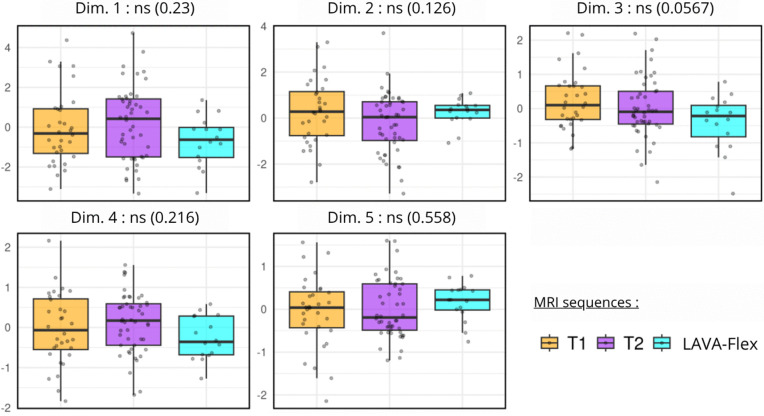
Coordinates of patches in the different PCA dimensions by MRI type. ANOVA and Welch’s ANOVA were performed on the different PCA dimension, based on the homoscedasticity criteria. P-values from ANOVA (Dims 1, 2, 4, 5) and Welch’s ANOVA (Dim 3) tests are displayed in the subplot titles. No significant (ns) differences between MRI types were observed.

Fig 4 shows that MRI type seems to have no impact on the position in the PCA plane. None of the ANOVA tests in any of the five PCA dimensions were significant when comparing the distributions of the MRI types. This result should be interpreted with caution, as the absence of a significant effect may reflect the limited sample size rather than a true lack of association.

**Fig 5:**
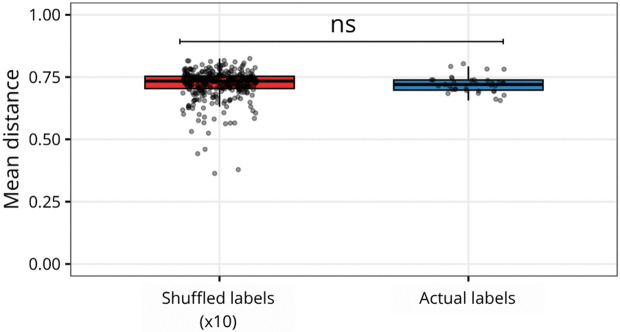
Intra-patient distance in seven dimensions compared to distances between random patches. The same data were used for the blue and red boxplots. For the shuffled labels, the patient identifiers associated with each MRI patch were randomly reassigned. This shuffling was repeated ten times to obtain a robust estimate. A Mann–Whitney test was performed to test for bias, which was not significant (ns) with a p-value = 0.113.

Regarding the patient effect, the dataset was built by selecting four patches per patient, two with lesions and two without, resulting in multiple slices per patient. To quantify this effect, we computed the mean distance between the different points in the PCA plane for each patient. To obtain a meaningful comparison, we shuffled the patient labels and again computed the average distance between points for the same patient. Fig 5 shows that the average distance between points from the same patient is not significantly smaller than the average distance obtained after random shuffling (ten shuffles were performed in Fig 5) (p-value = 0.113). In summary, our analysis shows that the presence or absence of a lesion is the main driver of the position in the PCA plane, and that neither the MRI sequence type nor the patient effect plays a significant role in explaining the projection.

**Fig 6:**
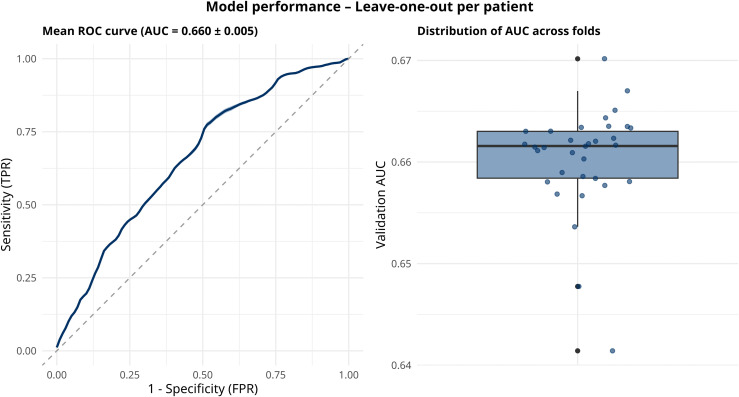
Predictions on validation dataset of the models trained with the Leave-one-out approach. Each of the 33 models trained during the Leave-one-out approach has been used to make predictions on the validation dataset. On the figure we can observe the mean ROC curve over the 33 models and the distribution of the 33 AUCs.

To provide evidence supporting the hypothesis of the absence of patient influence on the computed scores, we performed a leave-one-patient-out approach. For each patient in the proof-of-concept dataset, we removed that patient from the training step of the logistic regression, resulting in 33 trained models. Predictions were then made on the validation subset using these 33 models. Results are shown in Fig 6. The hypothesis was that if a patient included during the training step influences the results on its own images in the validation subset, performance should decrease across models and compared to the predictions obtained with the model trained on all patients from the PoC subset.

The mean AUC across the 33 models was 0.660 with a standard deviation of 0.005. The model trained on all 33 patients achieved an AUC of 0.667 on the validation subset, which is very similar to the results obtained with the leave-one-out approach.

### Asymmetry scores for automatic lesion detection inside a MRI patch

Once the scores were selected and the MRI type and patient bias were checked, we attempted to predict whether a lesion was present in a patch by using the computed asymmetry scores.

**Fig 7:**
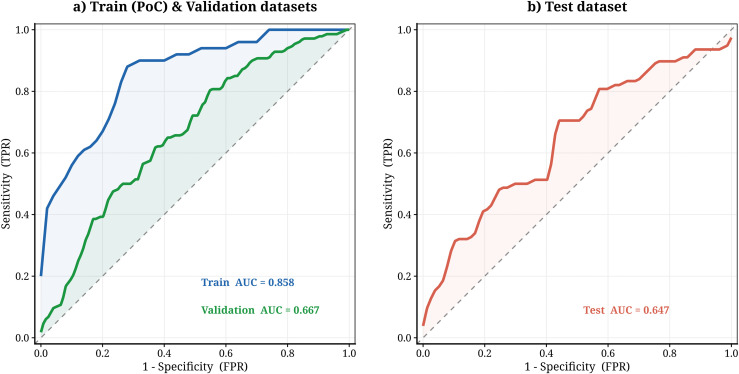
Results of training the logistic regression model and predicting the presence or absence of lesions in patches. (a) Training results of the logistic regression model on the PoC dataset.(blue curve) The AUC of 0.858 indicates good classification performance between labels in the Proof-of-Concept dataset. The results of the prediction on the validation dataset is shown with the green curve. (b) The model trained on the PoC dataset is applied to classify lesion or lesion-free patches in the test dataset.

To evaluate the ability of the model to generalize from clear lesion cases to more challenging slices, a logistic regression model was trained on the Proof-of-Concept (PoC) dataset and then evaluated on the validation dataset (new slices from the same patients) and testing dataset (slices from independent patients). Results are presented in Table 2 and Fig 7. The suitability of this approach was also confirmed by K-fold cross-validation (K = 5) at the patient level on the combined PoC and validation datasets, yielding fold-level AUCs ranging from 0.645 to 0.819 (mean AUC = 0.712, SD = 0.068), indicating moderate discriminative performance and variability across folds.

**Table 2:** Confusion matrices for logistic regression predictions on each dataset.

Dataset	True Label	Predicted Lesion-free	Predicted Lesion
Proof-of-Concept	Lesion-free	39	11
	Lesion	13	37
Validation	Lesion-free	85	39
	Lesion	67	73
Test	Lesion-free	50	27
	Lesion	38	40

Each dataset shows the number of patches correctly and incorrectly classified as ‘Lesion-free’ or ‘Lesion’.

Table 2 shows the confusion matrix of the model applied to the PoC dataset (which was also used for training). This resulted in a precision of 0.771, an accuracy of 0.76, a recall of 0.74, and an F1 score of 0.755. Overall, these results indicate that our deliberately simple approach (using a few interpretable scores applicable to multiple MRI types) does not lead to overfitting when trained on the PoC dataset.

Fig 7 illustrates the trade-off between sensitivity and specificity across different classification thresholds. The curve shows the model’s ability to discriminate patches according to their labels, with an area under the curve (AUC) of 0.858. This value indicates that, on average, the model assigns a higher score to a randomly selected slice with a lesion than to a randomly selected slice without a lesion in 85.8% of cases, confirming the good performance observed in the other evaluation measures.

We then used the trained logistic regression model to make predictions on the complete dataset without retraining. The objective was to determine whether the characteristics of the lesions identified by the model in the PoC dataset were similar to those in the complete dataset.

The PoC dataset consists of well-identified lesions that the model learned to detect based on the seven asymmetry scores. This training provides a score signature profile of what is considered a lesion. We then tested whether the same signature profile would still apply to less visible lesions. To address this question, we applied the model without retraining (transfer learning) to the validation dataset. Table 2 shows the confusion matrix of the model applied to the validation dataset. This resulted in a precision of 0.65, an accuracy of 0.598 and a recall of 0.571, indicating that the model struggled to identify whether a patch contained a lesion. As a result, the F1 score was 0.608, compared to 0.755 for the PoC dataset. As shown in Fig 7 (green curve), the corresponding area under the ROC curve (AUC) was 0.667, indicating that the logistic regression model ranked lesion patches higher than non-lesion ones in 66.7% of random comparisons.

Table 2 also shows that the model’s performance was notably affected by false negatives (67 lesions missed by the model). Upon further analysis of these patches, we found that 35 out of 67 were actually symmetric lesions, explaining why the asymmetry-based scores struggled to identify them.

Table 2 also presents the model’s performance on the test dataset, which included a large proportion of symmetrical lesions (*n* = 5 patients out of 11 in the test dataset). The proportion of symmetrical lesions in the test set (45%) is higher than the overall prevalence in the dataset (34.1%), but remains consistent with the variability expected from random sampling in a small cohort. The resulting metrics were an F1 score of 0.553, a precision of 0.568, an accuracy of 0.581, and a recall of 0.538. The corresponding AUC-ROC curve (red curve) is shown in Fig 7, with an AUC of 0.647. We observed an overall decrease in model performance on the test dataset, as also observed on the validation dataset.

To further analyze the prediction results on the test dataset, we computed classification performance at the patient level by aggregating slice-level predictions for each patient and calculating an accuracy score per patient. The resulting distribution of accuracies is consistent with the overall test set performance, with a median patient-level accuracy of 0.583.

### Identifying symmetric lesions based on asymmetry measures from all MRI types

**Fig 8:**
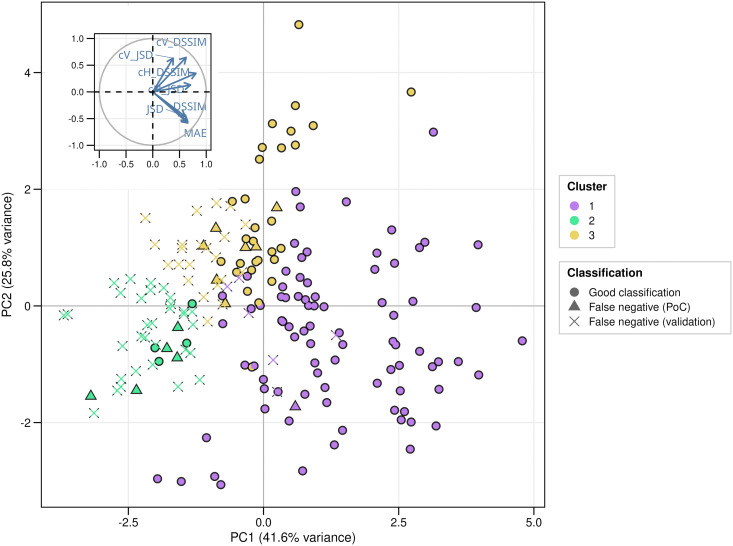
Cluster visualization on the PCA of the lesion slices of the combined PoC and validation datasets. PCA colored according to the clusters obtained from hierarchical ascending classification applied to the seven asymmetry measures of all patches presenting a lesion. Misclassification errors of the logistic regression are indicated with different shapes: triangles for false negatives in the Proof-of-Concept dataset and stars for false negatives in the validation dataset.

Among the possible shapes of anoperineal lesions, “horseshoe”-shaped lesions stand out from “classical” lesions due to their symmetry around the anterior–posterior axis. The fact that symmetric lesions are harder to detect can be illustrated by projecting their asymmetry scores along with those of the asymmetric lesions. Fig 8 shows the PCA projection of all lesion patches from the combined PoC and validation datasets. The correlation circle indicates that lesions with asymmetry higher than average are located on the positive side of the x-axis, while lesions with asymmetry lower than average are located on the negative side.

As an illustration, a hierarchical ascending classification was performed on the seven asymmetry scores to build clusters of patches. The optimal number of clusters was determined using silhouette analysis, which indicated three clusters. Each patch was then associated with its cluster using a color on the PCA projection, and each patch was annotated with a different marker depending on whether it was misclassified as a false negative during the predictions described in Section “Asymmetry scores for automatic lesion detection inside a MRI patch” (stars for misclassifications in the validation dataset, triangles for the PoC dataset). We observed that the majority of misclassified lesions were labeled in clusters 2 and 3. These two clusters are associated with lower asymmetry scores. By examining clusters 2 and 3 more closely, we identified the 28 symmetrical lesions that were misclassified as false negatives.

Finally, we note that, on average, errors in the PoC dataset (triangle shape) have higher coordinates on the x-axis than those in the validation dataset (star shape). This can be explained by the fact that lesions in the PoC dataset are well-identified, leading to higher average asymmetry scores. As a result, the decision threshold chosen by the model to distinguish lesion from non-lesion patches may be higher in the PoC dataset than in the complete dataset.

### Asymmetry scores enable locating lesions on a single MRI slice

**Fig 9:**
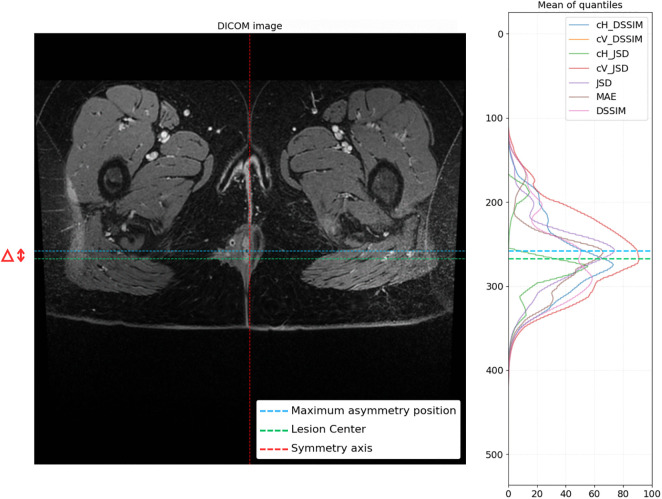
Identification of the Y-position associated with maximum asymmetry. A patch slides along the symmetry axis (red dotted line), pixel by pixel, and for each patch the seven asymmetry measures are computed. For each asymmetry measure, we select the corresponding percentile from the reference control (Lesion-free) distribution shown in Fig 2, resulting in a quantile value between 0 and 100 (right side of the figure). The position of maximum asymmetry is highlighted by the yellow dotted line, with the associated patch shown in blue. The actual position of the center of the lesion is indicated by the green dotted line. The distance between the center of the lesion and the maximum asymmetry is denoted as Δ.

Detecting a symmetric lesion proves to be a challenging task, as its asymmetry scores resemble those of non-lesion patches. However, such a lesion might still be detected within a slice as the “most asymmetric patch” relative to the rest. An illustrative example of an MRI slice with a lesion is shown in Fig 9.

To assess whether a lesion (including symmetric ones) can be detected within a slice, we applied a sliding patch along the y-axis of each slice in the different datasets (see Fig 9, left). For each position, we computed the asymmetry scores of the sliding patches. Each score value was then converted to a percentile value based on a reference distribution (the higher the score, the higher the corresponding percentile). To convert scores to percentile values, we used the distribution of non-lesion patches’ scores from the PoC dataset as the reference distribution.

We then retained the three highest percentile scores and computed their average, which we termed the 3-Peak Score. This produced a profile along the slice’s y-axis representing potential lesion detection (i.e., at least three locally high scores relative to the rest of the slice) (see Fig 9, right). We retrieved the position of the patch with the highest 3-Peak Score and compared it to the actual lesion position.

To do so, we computed the distance (in centimeters) between the center of this patch and the center of the lesion. We then assessed whether the localization performance of this approach was better than would be expected by chance. To generate the null distribution (H0), we randomly sampled patches along the slice and computed the distance between each random location and the actual lesion position. This yielded a baseline distribution against which our approach was compared.

We used two types of random sampling: one following a uniform distribution along the y-axis (restricted to the body area), and another following a beta distribution biased toward the posterior side of the body. This latter distribution captures a general biological bias observed in our dataset: lesions tend to occur toward the posterior side and are rarely located anteriorly. In fact, the majority of lesions are located primarily between the midline and the posterior part of the body. To account for this, we introduce a spatial weighting. Only the proof-of-concept dataset was used to estimate this distribution, ensuring no information from validation or test data was used. The distribution of lesion positions along the anteroposterior axis can be approximated by a beta distribution with parameters *a* and *b* using a method-of-moments approach, and defining the interval as the anteroposterior axis of the human body (*[start, end]*). Thus, the weights obtained outside the anatomical region (here, the patient’s body) are set to 0 and follow the distribution observed inside, which allows us to track the prediction probability provided by the prior. Consequently, we fitted a beta-like distribution to the lesion positions in the PoC dataset and used this as “prior knowledge” for our 3-Peak score and a random sample.

**Fig 10:**
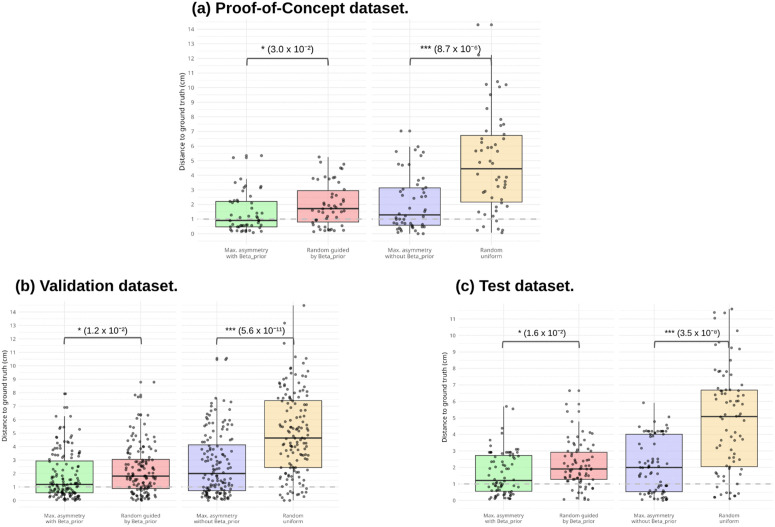
Distance between lesion position and maximum-asymmetry patch in the different datasets. The maximum-asymmetry approach incorporating the beta prior is compared to random sampling guided by the same beta prior, while the maximum-asymmetry method without the beta prior is compared to uniform random sampling. A Mann–Whitney test shows that the method using the beta prior performs significantly better than random sampling, and the method without the beta prior performs better than uniform random sampling.

Fig 10a shows the distributions of distances between predicted and actual lesion localizations in the PoC dataset. Approximately 45–50% of the predicted positions are within 1 cm of the actual lesion (see dotted line), both with and without applying the beta bias (green and blue distributions, respectively). Overall, the figure demonstrates that our approach outperforms both random sampling methods (uniform and beta-biased). Finally, including the beta bias improves the localization accuracy of the best patch relative to the ground truth.

Figs 10b and 10c show the corresponding distributions obtained by applying the method to the validation and test sets, respectively. The same conclusions hold: our approach outperforms random sampling, and adding a beta bias further improves the automatic localization of the lesion. The proportion of symmetric lesions in the test dataset does not reduce the performance of our method. A possible explanation is that even when the lesion is symmetric, it remains the most asymmetric area of the MRI slice.

## Discussion and future directions

In this study, our objective was to investigate anoperineal lesions in axial pelvic MRI sequences in the context of a small sample size and diverse MRI types, a situation that often reflects actual clinical practice [8,17–19]. This led us to introduce asymmetry-based measures to ensure both interpretability and robustness across various types of MRI. The underlying assumption is that the presence of a lesion generates local asymmetry in the MRI signal that exceeds the natural asymmetry of the human body in MRI.

As a first step to validate this assumption, we applied well-known asymmetry measures to MRI slices presenting or not presenting lesions. We showed that seven of these measures were sensitive to the presence of lesions, even in the context of limited data and heterogeneous MRI sequences. Moreover, we showed that these scores seems not to be biased by patient effects (Fig 5) or by MRI sequence type (Fig 4). Further analyses on larger cohorts would be needed to confirm this result.

Then, based on these seven scores, we developed an automatic classifier to assess the presence of a lesion. By construction, we anticipated that the approach would be less efficient in cases of symmetrical or quasi-symmetrical lesions. Interestingly, despite this limitation and the inherent complexity of pelvic tissue representation in MRI, our results demonstrate that asymmetry measures can capture lesion-related patterns (Fig 7). The use of logistic regression was particularly suitable due to its minimal number of fitted parameters, ensuring both interpretability and adequacy for small datasets. The logistic regression model produced encouraging results, demonstrating that satisfying predictive performance was maintained on the validation and test datasets, for a precursor study, despite being trained on a reduced portion of the data.

Similarly, the task of localizing symmetrical lesions is equally challenging. Nonetheless, we demonstrated that asymmetry scores were surprisingly effective for localizing lesions within a slice, even in the case of symmetrical lesions (Fig 10c). Approximately 50% of lesions were detected within one centimeter of their center. This is notably true in the test dataset, which was composed of 45% of symmetrical lesions. Even for asymmetric lesions, lesion localization remains a challenging task due to the heterogeneity in size, diameter, and shape of perianal lesions. We defined the lesion position as the center of its vertical range on the slice. In the case of irregularly shaped or extensive lesions, this position may not correspond to the area of maximum morphological asymmetry; therefore, we did not expect a perfect match between the maximum asymmetry score and the lesion center. However, our method still demonstrates strong localization performance across all datasets: Proof-of-Concept, Validation, and Test. This approach can be viewed as a proof of concept demonstrating the relevance of asymmetry measures for detecting perianal lesions. The use of larger datasets could enable the development of more sophisticated methods incorporating these features.

Across both classification and localization tasks, high asymmetry scores consistently correspond to the presence of lesions. This is a strong result, as it confirms the initial assumption that asymmetry reflects pathological lesions rather than normal anatomical variations. The fact that elevated scores align with actual lesions demonstrates that our method captures pathological signals beyond natural morphological asymmetry.

A major limitation of this study is the small size of the dataset and the lack of truly healthy controls. To compensate for this, we defined “control” slices as MRI slices located just outside the lesion area but still within the perianal region. The use of adjacent slices as controls raises the possibility that subtle tissue displacements caused by nearby lesions may produce low-level asymmetry. We further investigated whether asymmetry measures vary with the distance between lesion-free slices and the lesion center. The analysis showed no meaningful relationship between distance and asymmetry scores, suggesting that control slices can be used consistently regardless of their proximity to the lesion. However, we cannot guarantee that the asymmetry levels observed in these lesion-free slices are comparable to those of truly healthy subjects. This may introduce a slight bias in both classification and localization tasks and would require further validation using datasets including healthy controls.

The dataset also includes a broad variety of MRI sequences, encompassing multiple acquisition protocols with distinct contrasts and specificities. Despite this variability, asymmetry-based scores consistently captured lesion-related patterns, regardless of sequence type. As a result, our logistic regression model enabled robust lesion detection across T1-weighted, T2-weighted, LAVA-FLEX, diffusion-weighted, and ADC images.

Our method also proved resistant to MRI artifacts such as shadow bands and zebra stripes, which can introduce noise and reduce localization accuracy [28–33]. Since the scans were performed at multiple centers with different equipment, not all artifacts could be corrected [34,35]. Despite this, lesions consistently altered the distribution of asymmetry scores, demonstrating that our method detects meaningful structural signals beyond imaging artifacts. This confirms the reliability of our approach and provides a solid foundation for further improvements.

Defining a tolerance criterion for lesion localization is difficult due to the diversity in lesion size and shape. Our method measures the distance from the lesion core, but this core can range from a small, round spot to a large, diffuse area, complicating performance evaluation. We also aimed to investigate whether lesion severity correlates with higher asymmetry scores by comparing our results with established clinical indices such as the Van Assche score [36] and MAGNIFI-CD [37]. We considered investigating potential relationships between asymmetry measures and clinical indices such as the Van Assche and MAGNIFI-CD scores. However, the limited availability of these annotations in our cohort prevented any formal correlation analysis. Nonetheless, several theoretical links can be proposed. For instance, a higher number of fistulous tracts could increase the spatial extent and structural complexity of lesions on MRI slices, potentially leading to higher wavelet-based asymmetry scores due to more irregular, multidirectional patterns. Similarly, T2 hyperintensity, reflecting active inflammation, may be associated with increased asymmetry in raw intensity-based measures, as the contrast between lesion and surrounding tissue accentuates lateral imbalance. Finally, the presence of inflammatory masses or accumulations could result in a general increase in asymmetry indicators due to greater lesion extent and heterogeneity. These hypotheses would require validation on larger cohorts with systematically annotated clinical scores.

Although our study was conducted on a relatively small dataset, the asymmetry-based measures we present open promising avenues for future research. If larger, dedicated cohorts become available, these measures could serve not only as standalone indicators but also as robust and interpretable inputs for more advanced machine learning models. Such integration could enable the development of predictive systems capable of capturing subtle and complex patterns that escape human interpretation. In the long term, extending this approach to larger populations could support the development of clinically relevant decision-support tools, thereby improving early detection, monitoring, and management of Crohn’s disease-related lesions.
