# Supplementary material for: Sequence-robust MRI asymmetry measures for the detection of anoperineal lesions in Crohn’s disease on axial pelvic MRI scans
Source: PLoS One. 2026 Jul 21;21(7):e0340243. doi: 10.1371/journal.pone.0340243 (PMC13387581; doi:10.1371/journal.pone.0340243)
Supplement: Attachment — Submitted filename: Recommendation.pdf [file pone.0340243.s001.pdf]

The authors present a computational method to detect and localize anoperineal lesions in Crohn's disease using axial pelvic MRI scans. Addressing the limitations of deep learning on small, heterogeneous medical datasets, the authors utilize a simpler, interpretable approach based on asymmetry measures (JSD, MAE, DSSIM) combined with Gaussian filtering and wavelet transforms. Evaluated on a cohort of 44 patients (134 MRI sequences), the logistic regression classifier achieved an AUC of 0.858 on a curated Proof-of-Concept (PoC) dataset, but dropped to an AUC of 0.594 on a test set containing symmetrical lesions. However, the localization task showed promise, identifying the lesion within roughly 1 to 1.2 cm of its actual center across datasets

1. The PoC dataset explicitly excludes symmetrical lesions to prove that the asymmetry features work. However, the test set includes a highly disproportionate number of symmetrical lesions (4 out of 9 patients).
2. While this transparently explains the performance drop (AUC 0.858 to 0.594), it creates a jarring narrative regarding the model's clinical viability. Training on a dataset that intentionally filters out a specific phenotype, and then testing on a dataset heavily skewed by that phenotype, is an experimental design flaw.
3. Author must provide the overall prevalence of symmetrical (e.g., horseshoe) lesions within the full 3TLAP cohort. Is 4/9 representative of the actual clinical distribution, or an artifact of an unfavorable random split? If it is an artifact, the test set should be resampled via cross-validation across the entire 44-patient cohort to provide a more accurate representation of generalized performance.
4. The authors state that lesion-free control slices were taken from the perianal area of the *same* patients, outside the lesion range. In the discussion, you acknowledge this is a major limitation as nearby lesions may cause tissue displacement and low-level asymmetry. This is a critical methodological vulnerability. By definition, your controls are not truly healthy, and the baseline asymmetry distribution used to calculate the 3-Peak Score quantiles is inherently contaminated.
5. The manuscript must explicitly justify why scans from genuinely healthy controls were not obtained (e.g., ethical constraints, retrospective cohort limitations). Furthermore, an analysis quantifying the distance between the control slices and the lesion slices should be added to see if low-level asymmetry decays the further the control slice is from the lesion range.

6. The Data Availability statement mentions that data is available from the CHU of Rennes Institutional Data Access, listing Guillaume Bouguen as the contact. PLOS One policy typically requires a non-author institutional point of contact for data requests to ensure long-term access and prevent conflicts of interest. Guillaume Bouguen is listed as an author on this paper.
7. Please provide an institutional email address or an ethics committee contact that is independent of the authoring team for data requests.
8. The abstract must explicitly state that the classification model struggles significantly with symmetrical lesions, as the AUC drop on the complete dataset is heavily tied to this limitation.
9. In Section 2.3.2, the correlation formula is provided . Please ensure standard mathematical typesetting is utilized in the final manuscript submission and define exactly what pixel intensity ranges are used for X_i and Y_i.
10. **Figure 2:** The adjusted p-values are listed using scientific notation (e.g., 3.6e-7). It is generally better formatting to use standard scientific notation (e.g., 3.6×10^{-7}) in publication-grade figures.
11. In the discussion, you mention an attempt to correlate asymmetry scores with clinical indices like the Van Assche score and MAGNIFI-CD, but note that too few medical reports included these values. You should briefly hypothesize *how* an asymmetry score might theoretically map to these indices (e.g., does a higher JSD correlate to more extensive tract branching?).
