# Supplementary material for: Sequence-robust MRI asymmetry measures for the detection of anoperineal lesions in Crohn’s disease on axial pelvic MRI scans
Source: PLoS One. 2026 Jul 21;21(7):e0340243. doi: 10.1371/journal.pone.0340243 (PMC13387581; doi:10.1371/journal.pone.0340243)
Supplement: Attachment — Submitted filename: Response_to_reviewers.pdf [file pone.0340243.s003.pdf]

We thank the reviewers for their insightful comments and suggestions, which have helped improve the quality and clarity of the manuscript.

Reviewer #1:

1. The study looks at an important clinical problem and the general idea makes sense. Using left–right asymmetry in MRI scans to detect lesions is logical, and the method is clearly explained. The early analyses show that some of the asymmetry measures are higher when lesions are present, and the localization results suggest that the approach could be useful in practice. However, the conclusions feel a bit stronger than what the data can fully support. The study only includes 44 patients, and the independent test group has just nine patients, which is quite small. This makes it hard to be confident that the method would work equally well in other hospitals or larger populations.

We agree that the relatively small size of our cohort is a limitation of this study, and we have revised the manuscript to moderate our conclusions accordingly. In addition, following the reviewer's suggestions, the data partitioning strategy, which results in an updated independent test set comprising 11 patients instead of 9. While this remains limited, we now more explicitly acknowledge this constraint and frame our findings as preliminary, pending validation on larger and more diverse cohorts.

2. Also, the Proof-of-Concept dataset was created using very clear and mostly asymmetric lesions, which likely made the method look better during development. When the model was applied to the full dataset and the test set, performance dropped, which shows that the real-world situation is more challenging. Overall, the results show that the idea is promising, but the claims about robustness, independence from MRI type, and clinical usefulness should be more cautious, since this is still an early-stage study with limited data.

We thank reviewer 1 for this important comment. We agree with the reviewer that our dataset split was not fully convincing and enough explained. As detailed below, we have:

- Reorganized and clarified the patient split.
- Performed a "Leave-one-out" approach at the patient level.
- Performed a cross-validation at the patient level.

3. The statistical methods used in the paper are reasonable for exploring the data, but they are not strong enough to fully support firm conclusions. The authors used appropriate tests like nonparametric comparisons, ANOVA, and PCA to look at patterns in the data, which is fine for exploratory work. However, the way the prediction model was evaluated is not fully convincing. It is not clearly shown whether proper cross-validation was done, especially at the patient level, raising concerns about optimistic bias and potential within-patient dependence among slices.

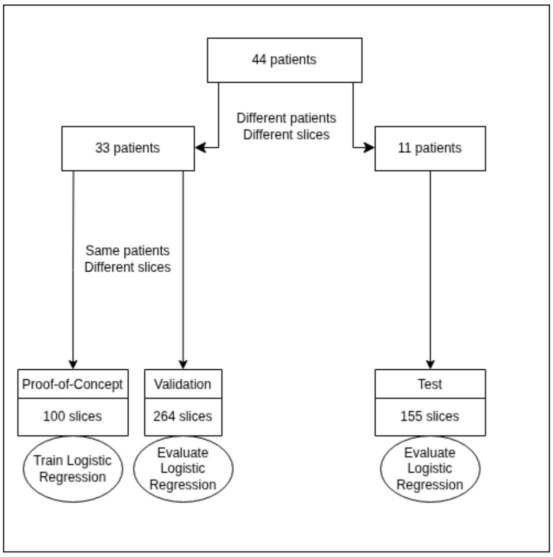

We thank Reviewer 1 for this pertinent comment. We agree that the sample size, particularly the size of the independent test set, limits the scope of our conclusions and the generalizability of the results to other centers or larger populations.

To clarify the patient split, we decided to include in the validation dataset only those patients who were part of the proof-of-concept dataset. Since these patients were not used for score selection or for training the logistic regression model, we transferred these two patients from the validation dataset to the test dataset.

We now define three distinct datasets that address different questions:

- The proof-of-concept dataset (100 slices) includes slices from 33 patients displaying clear asymmetrical lesions. This dataset is used to select metrics sensitive to asymmetry and to train the classifier.
- The validation dataset (264 slices) includes different slices from the same 33 patients as in the proof-of-concept dataset (centered on the lesion range or outside this area but still within the perineal region). This dataset is used to investigate how the model behaves in more realistic conditions. In the revised version, this dataset is also used to assess patient effects using a leave-one-out approach.
- The test dataset (155 slices) has been slightly enlarged in the revised version and now contains slices from 11 patients, none of whom are included in the 33 patients from the proof-of-concept or validation datasets. This dataset is used to evaluate generalization to new patients.

We have revised the Methods section to clarify the definition and role of the three datasets. We have rerun the models and the localization task and updated the Results section accordingly. We also added an analysis of patient effects using a leave-one-out approach. Although the main conclusions remain

unchanged, we agree that their formulation was initially too strong and have moderated them in the revised version.

4. Since multiple MRI slices come from the same patient, treating them as completely independent could make the results look better than they really are.

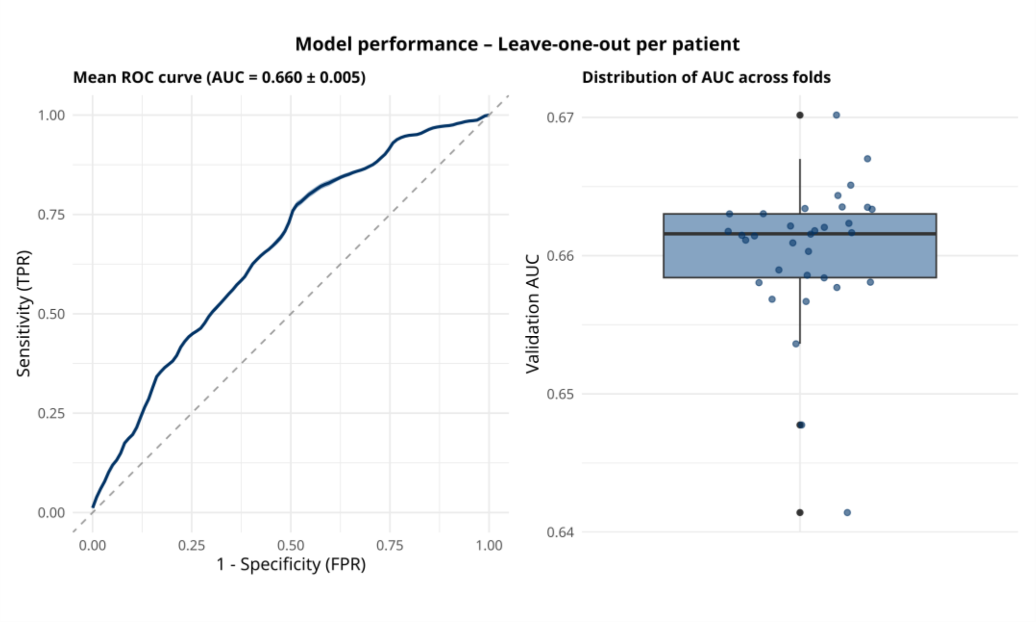

We acknowledge that the statistical analyses conducted in this article are primarily exploratory in nature and do not support definitive conclusions. To address this concern, we implemented two actions: we clarified the patient-level data partitioning, and we performed a leave-one-patient-out analysis.

Regarding the evaluation of the prediction model, we aimed to more explicitly assess the independence of the scores from individual patients. To this end, we used the validation subset to evaluate the stability of model performance when a patient is excluded from logistic regression training (leave-one-out procedure). The validation set contains the same 33 patients as the training set, but with different images.

The underlying hypothesis is that if model performance depends strongly on the presence of a given patient in the training set, then predictive performance should vary significantly when that patient is excluded. In total, 33 models were trained and evaluated on the validation dataset. The results show good performance stability, with a standard deviation of 0.005 for the AUC on the validation set, and AUC values ranging from 0.64 to 0.67. This suggests that the model is not highly sensitive to the inclusion or exclusion of any single patient during training.

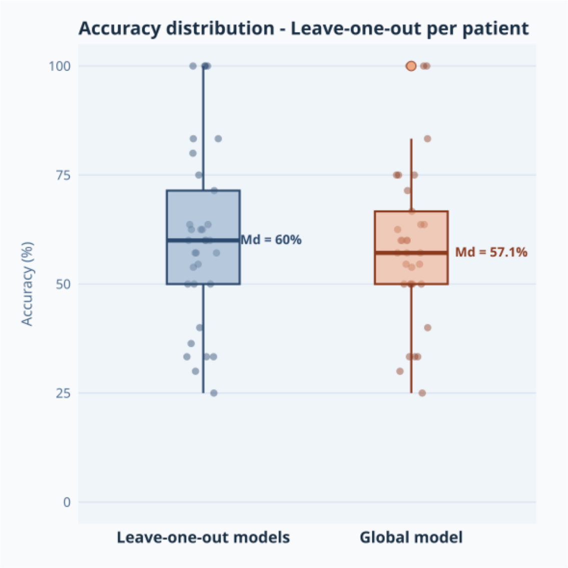

We also examined the performance of the 33 models on images from the patient excluded during training. For each iteration, the model trained without a given patient was used to predict that patient's images in the validation set. The results show no significant difference between the leave-one-out models and the model trained on the complete proof-of-concept dataset (median accuracies: 0.60 and 0.571, respectively). These findings do not suggest a systematic loss of accuracy when a patient is removed from the training set for logistic regression.

5. The paper also does not report confidence intervals for key performance measures like AUC, precision, recall, or F1-score, so it is hard to judge how stable or uncertain the results are. The independent test set is also very small, which makes it difficult to draw strong conclusions about how well the model would perform in other settings.

Regarding confidence intervals, we address this comment more precisely in question 8 (patient-level cross-validation).

Regarding the size of the independent test set, we increased the proportion of patients assigned to the test set from 20% to 25%. We agree that it nevertheless remains relatively small (11 patients). Accordingly, we have moderated our conclusions where appropriate and now explicitly acknowledge this limitation in the Discussion section.

6. In addition, the authors interpret non-significant results about MRI type as proof that MRI type has no effect, but with such a small sample, it may simply be that the study does not have enough power to detect differences.

We agree with reviewer 1 on this statement and we mitigated our conclusions in the revised version. The title has also been changed to go along with the modifications within the text.

7. Overall, the statistical approach needs stronger validation methods and clearer reporting to make the findings more reliable.

We hope that the additional methodological steps we have implemented - namely, the reorganization and clarification of patient-level data partitioning, the application of a leave-one-out analysis at the patient level, and the introduction of cross-validation procedures - adequately address the reviewer's concerns.

8. To enhance the statistical rigor of the manuscript, the authors should implement patient-level cross-validation. This approach would ensure that the model's performance is evaluated based on how well it generalizes to new patients, rather than just relying on the same data used for training.

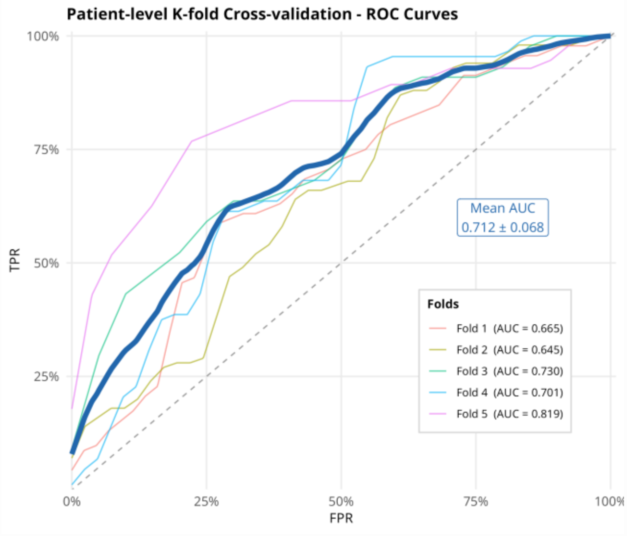

As suggested, we performed a patient-level *k*-fold cross-validation on the combined proof-of-concept and validation datasets. The AUC values obtained across the different folds range from 0.645 to 0.819, with a mean AUC of 0.712 (standard deviation = 0.068).

In comparison, the model trained on the proof-of-concept dataset alone shows similar performance when evaluated on the validation and test sets, with AUC values of 0.667 and 0.647, respectively. Overall, the results of the patient-level cross-validation are consistent with those observed on the independent test set.

9. Additionally, the authors should provide bootstrapped confidence intervals for their performance metrics, such as AUC, precision, recall, and F1-score.

The revised version of the manuscript now includes confidence intervals derived from the leave-one-out analysis and the patient-level cross-validation. We are unfortunately unable to provide confidence intervals for the independent test set, as it consists of a single, fixed cohort of patients.

10. To make the results more reliable, the authors should use stronger validation methods, such as checking performance at the patient level rather than at the slice level, and reporting confidence intervals.

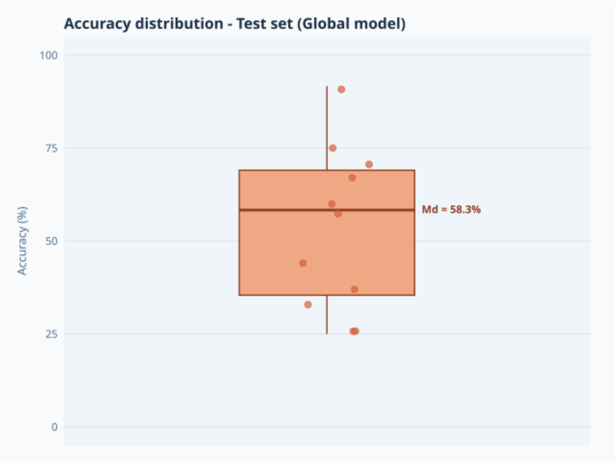

Patient-level classification results are illustrated in this boxplot. For each patient in the test subset, we used the logistic regression model trained on the proof-of-concept dataset to classify their images. We then constructed a confusion matrix for each patient and computed the corresponding accuracy. The results are comparable to those obtained on the validation dataset, with a median accuracy of 58.3%. The overall accuracy on the test dataset is 58.1%, which is consistent with these patient-level results.

11. They should also clearly explain their assumptions about independence in the data. These steps would make the statistical conclusions more solid and trustworthy.

Our assumptions regarding independence primarily concern the structure of the data at the patient and image levels. In particular, multiple MRI slices are acquired from the same patient and cannot be considered fully independent observations.

To address this, we explicitly designed our evaluation strategy to account for within-patient dependence. First, the independent test set is defined at the patient level, ensuring that no patient contributes to both training and test sets. Second, we evaluated the stability of the model using a leave-one-out analysis at the patient level, which assesses the impact of removing an entire patient from the training set. This analysis shows stable performance (AUC standard deviation = 0.005), suggesting limited sensitivity to individual patients. In addition, we report patient-level performance metrics (by aggregating slice-level predictions), which provide a more appropriate assessment of model behavior in the presence of correlated observations.

As noted by Reviewer 3, the main remaining assumption is that control patches can be obtained from slices outside the lesion range of patients. This point is now explicitly discussed in the revised manuscript.

Reviewer #2:

The manuscript tackles a clinically important and underexplored problem—automated assessment of anoperineal/perianal Crohn's lesions on axial pelvic MRI—with an interpretable, anatomy-aligned asymmetry pipeline designed for small, heterogeneous cohorts. The workflow estimates a bilateral symmetry axis, applies Gaussian denoising and a Haar wavelet transform, then computes engineered left–right asymmetry measures for (i) patch classification via a logistic regression model and (ii) within-slice localization using a sliding window and a "3-Peak Score" (average of the top three normalized asymmetry scores) to identify the most likely lesion position along the y-axis. The emphasis on interpretability is a clear strength, and the inclusion of a localization task is a meaningful step toward workflow support rather than detection alone.

1. That said, parts of the current evaluation risk optimistic estimates and therefore weaken the strongest claims. In particular, the PoC dataset is used to select the "significant" asymmetry measures and to train the logistic regression model, and performance is also reported on that same PoC set. Please label this explicitly as resubstitution/proof-of-concept performance and complement it with patient-level cross-validation (ideally nested if feature selection and normalization steps are repeated) or a proper internal validation split, while keeping the held-out test patients fully untouched.

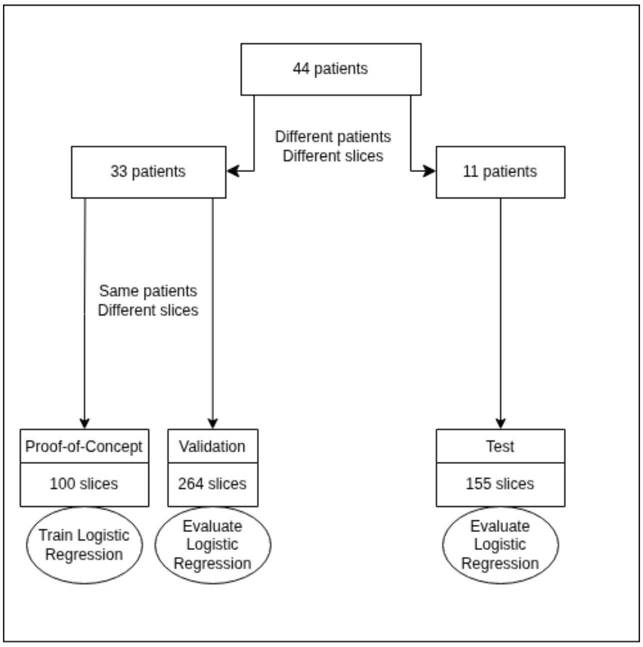

We thank Reviewer 2 for this pertinent comment, which was also raised by Reviewer 1. We provide a joint response to both comments below.

We agree that the sample size, particularly the size of the independent test set, limits the scope of our conclusions and the generalizability of the results to other centers or larger populations.

To clarify the patient split, we decided to include in the validation dataset only those patients who were part of the proof-of-concept dataset. Since these patients were not used for score selection or for training the logistic regression model, we transferred these two patients from the validation dataset to the test dataset.

We now define three distinct datasets that address different questions:

- The proof-of-concept dataset (100 slices) includes slices from 33 patients displaying clear asymmetrical lesions. This dataset is used to select metrics sensitive to asymmetry and to train the classifier.
- The validation dataset (264 slices) includes different slices from the same 33 patients as in the proof-of-concept dataset (centered on the lesion range or outside this area but still within the perineal region). This dataset is used to investigate how the model behaves in more realistic conditions. In the revised version, this dataset is also used to assess patient effects using a leave-one-out approach.
- The test dataset (155 slices) has been slightly enlarged in the revised version and now contains slices from 11 patients, none of whom are included in the 33 patients from the proof-of-concept or validation datasets. This dataset is used to evaluate generalization to new patients.

We have revised the Methods section to clarify the definition and role of the three datasets. We have rerun the models and the localization task and updated the Results section accordingly. We also added an analysis of patient effects using a leave-one-out approach. Although the main conclusions remain unchanged, we agree that their formulation was initially too strong and have moderated them in the revised version.

As suggested, we additionally performed a patient-level k-fold cross-validation on the combined Proof-of-Concept and validation datasets. The AUCs obtained across the different folds range from 0.645 to 0.819, with a mean AUC of 0.712 (Standard deviation = 0.068). In comparison, the model trained on the Proof-of-Concept dataset alone exhibits similar performance on the validation set and the test set, with AUCs of 0.667 and 0.647, respectively. The results of the patient-level cross-validation are similar to those observed with our independent test set.

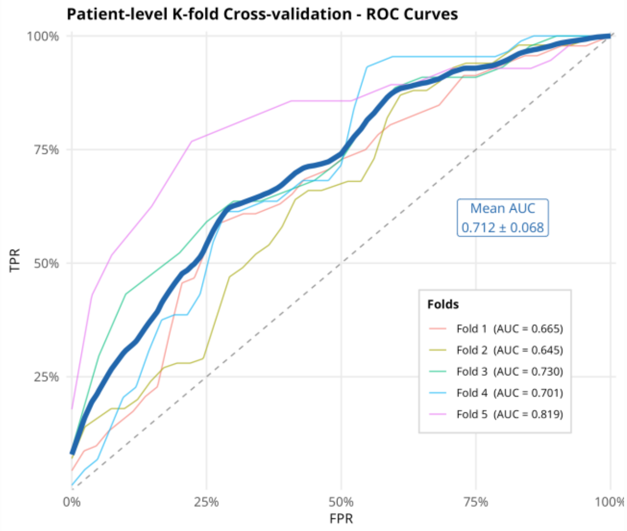

2. Relatedly, please address the dependence structure: the dataset contains multiple sequences and slices per patient, yet key metrics are presented at the patch level as if observations were independent. Even with the "patient effect" analysis in PCA space, the classification and localization results would be more convincing with patient-level reporting and cluster-aware uncertainty.

This comment partially overlaps with comment 11 from Reviewer 1, and we provide a unified response.

Following the reviewers' comments, we have clarified the construction of our datasets and explicitly accounted for the dependence structure induced by multiple slices per patient. In particular, we now distinguish between: (i) independent images from the same patients (validation dataset), and (ii) independent images from independent patients (test dataset). The independent test set is defined at the patient level, ensuring that no patient contributes data to both training and test sets.

To further assess the impact of within-patient dependence, we implemented two complementary analyses. First, we performed a leave-one-out analysis at the patient level. Using the validation dataset (which contains different images from the same 33 patients as the training set), we evaluated the stability of model performance when each patient is excluded from training. In total, 33 models were trained and evaluated. The results show stable performance, with AUC values ranging from 0.64 to 0.67 and a standard deviation of 0.005, suggesting limited sensitivity to any individual patient.

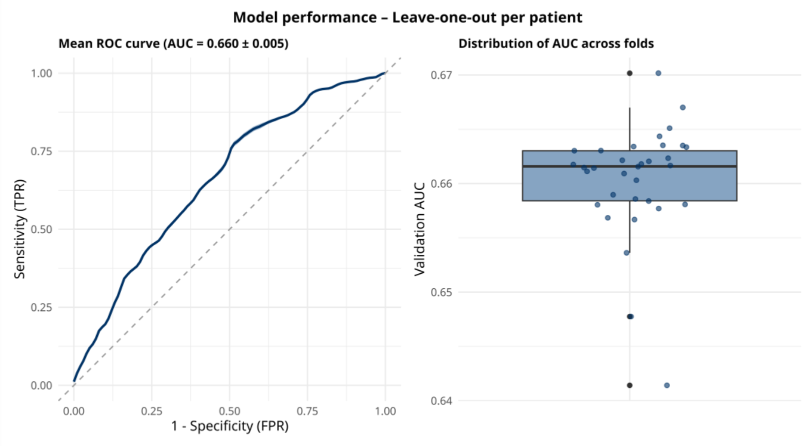

In addition, for each iteration of the leave-one-out procedure, the model was evaluated on images from the excluded patient. This analysis shows no significant difference compared to the model trained on the full proof-of-concept dataset (median accuracies: 0.60 vs 0.571), indicating no systematic loss of performance when a patient is removed from training.

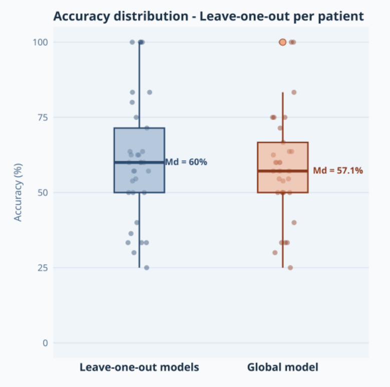

Second, we report patient-level classification performance by aggregating slice-level predictions. For each patient in the independent test set, predictions were computed on all slices and summarized through a confusion matrix to obtain patient-level accuracy. The resulting distribution (shown in the revised manuscript) exhibits a median accuracy of 58.3%, consistent with the overall test accuracy (58.1%) and with results observed on the validation dataset.

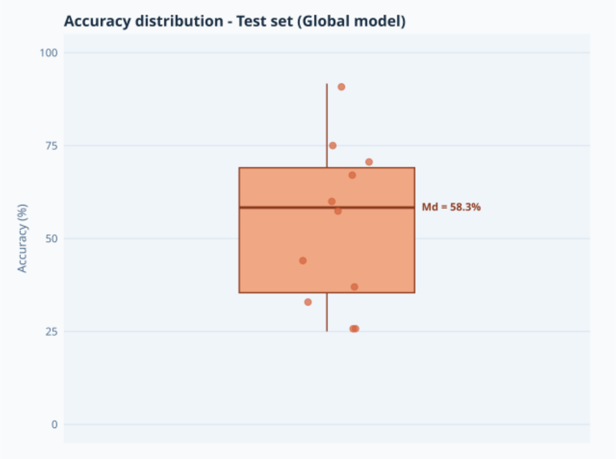

Overall, these analyses provide a more appropriate evaluation of model performance in the presence of correlated slice-level observations and help mitigate the risk of optimistic bias due to within-patient dependence. We acknowledge that this remains a proof-of-concept study and that further validation on larger, fully independent cohorts will be necessary.

3. The clinical framing and interpretation of test-set results also need strengthening. The held-out test classification performance is modest, so the manuscript would benefit from a sharper, concrete use case (triage, reader aid, or quality control) and at least simple baselines beyond the proposed asymmetry features.

We thank Reviewer 2 for this constructive comment. We have clarified in the revised manuscript that, given the observed performance levels (AUC ≈ 0.6–0.7), the proposed approach is not intended for standalone clinical use. Rather, the objective of this work is to demonstrate that asymmetry-based features capture meaningful information related to lesion presence.

In this context, our results support the idea that left–right asymmetry provides a relevant signal that could be leveraged in more advanced models. For instance, such features could be integrated as complementary, interpretable inputs in multimodal frameworks, or combined with deep learning approaches trained on larger MRI cohorts. More broadly, this work should be viewed as a proof of concept highlighting the potential of asymmetry-driven representations, rather than a fully developed clinical tool.

We have added a dedicated paragraph in the Discussion section to explicitly acknowledge these limitations and clarify the intended scope and potential applications of the proposed approach.

4. In addition, the test split contains a high proportion of symmetric (“horseshoe”) lesions (4/9 patients), which is described as an “unfavorable random split” and used to explain degraded classification performance. Because symmetry is an acknowledged failure mode for asymmetry-based detection, please frame this explicitly as a key limitation, and describe the splitting strategy clearly (and ideally use stratified or repeated patient-level splitting).

We explicitly acknowledge that the presence of symmetric lesions constitutes a key limitation of our asymmetry-based approach, as such cases represent an inherent failure mode of the method.

Regarding the data split, approximately 34.1% of patients in the full dataset present symmetric lesions. Given the limited cohort size, it is challenging to simultaneously enforce patient-level independence and stratification based on lesion symmetry. As a result, the proportion of symmetric lesions in the test set (5/11 patients, 45%) is higher but remains broadly consistent with the overall prevalence.

We have clarified the splitting strategy in the revised manuscript and now explicitly report the global prevalence of symmetric lesions in the Results section (“Asymmetry scores for automatic lesion detection inside an MRI patch”). We also emphasize in the Discussion that the presence of symmetric lesions can negatively impact performance and should be considered when interpreting the results.

5. The claim of MRI-type independence needs more direct support. The paper argues that scores are “not sensitive” to MRI type largely via PCA and (Welch’s) ANOVA on PCA dimensions, and then makes broader statements about sequence independence. If MRI-type independence is a central claim, please support it with stratified performance (or at least subgroup summaries) by sequence type, rather than relying on PCA/ANOVA alone.

We agree that our conclusions were too strong, and we mitigated our statements in the revised version. The title has also been changed to go along with the modifications within the text.

6. Please also improve reproducibility and method specificity. Patch sizes are given in centimeters (1 cm × 7 cm) and the localization window steps by one pixel, but the cm-to-pixel conversion across varying DICOM spacings is not fully specified; please add exact implementation details.

We thank the reviewer for this important comment regarding reproducibility. The patch dimensions were determined by analyzing the distribution of lesion distances relative to the median symmetry axis of the MRI, which yielded optimal sizes of 1 cm (height) × 7 cm (width).

The centimeter-to-pixel conversion is performed automatically for each DICOM MRI sequence using the image spacing provided in the metadata. Specifically, we retrieve the in-plane pixel spacing (in millimeters) from the DICOM header via the GetSpacing() function from the *SimpleITK* library. The conversion is then computed by multiplying the size in centimeters by 10 to obtain millimeters, and dividing by the pixel spacing (in millimeters) and then round up the result. For example, with a spacing of 0.5 mm per pixel, a width of 7 cm (i.e., 70 mm) corresponds to 140 pixels.

These implementation details have now been explicitly added to the Methods section to ensure full reproducibility of the cm-to-pixel conversion.

7. The beta-biased null for localization is described as a beta-like distribution fit to PoC lesion positions; please report how parameters are estimated and ensure this prior is fit only on training data.

As noted by the reviewer, this prior is estimated exclusively on the proof-of-concept (PoC) dataset. We have clarified this point in the Methods section to ensure that the prior is fitted only on training data.

The parameters α and β of the beta distribution are estimated using a method-of-moments approach, based on the empirical distribution of lesion positions along the anteroposterior axis in the PoC dataset. The interval is defined over the full body axis (from anterior to posterior) and normalized according to each patient's anatomical dimensions.

The key observation is that lesion locations are not uniformly distributed, with a higher concentration in the mid-to-posterior regions. To incorporate this anatomical prior, we apply a spatial weighting scheme: outside the anatomical region (i.e., outside the patient's body), the weight is set to zero; within the anatomical region, the weight corresponds to the value of the fitted beta density.

This approach allows us to model a bounded and asymmetric spatial distribution consistent with anatomical constraints, while preserving a strict separation between training and evaluation data.

8. Finally, please consider sharing code or derived features (even if raw data cannot be public due to privacy restrictions) to improve reproducibility.

We thank the reviewer for this important comment regarding reproducibility and open science. The code and example data will be made available on a public GitHub repository. While raw medical imaging data cannot be shared due to privacy constraints, we will provide derived data, including files containing the asymmetry scores computed from patient MRI scans.

Reviewer #3:

The authors present a computational method to detect and localize anoperineal lesions in Crohn's disease using axial pelvic MRI scans. Addressing the limitations of deep learning on small, heterogeneous medical datasets, the authors utilize a simpler, interpretable approach based on asymmetry measures (JSD, MAE, DSSIM) combined with Gaussian filtering and wavelet transforms. Evaluated on a cohort of 44 patients (134 MRI sequences), the logistic regression classifier achieved an AUC of 0.858 on a curated Proof-of-Concept (PoC) dataset, but dropped to an AUC of 0.594 on a test set containing symmetrical lesions. However, the localization task showed promise, identifying the lesion within roughly 1 to 1.2 cm of its actual center across datasets

1. The PoC dataset explicitly excludes symmetrical lesions to prove that the asymmetry features work. However, the test set includes a highly disproportionate number of symmetrical lesions (4 out of 9 patients). While this transparently explains the performance drop (AUC 0.858 to 0.594), it creates a jarring narrative regarding the model's clinical viability. Training on a dataset that intentionally filters out a specific phenotype, and then testing on a dataset heavily skewed by that phenotype, is an experimental design flaw.

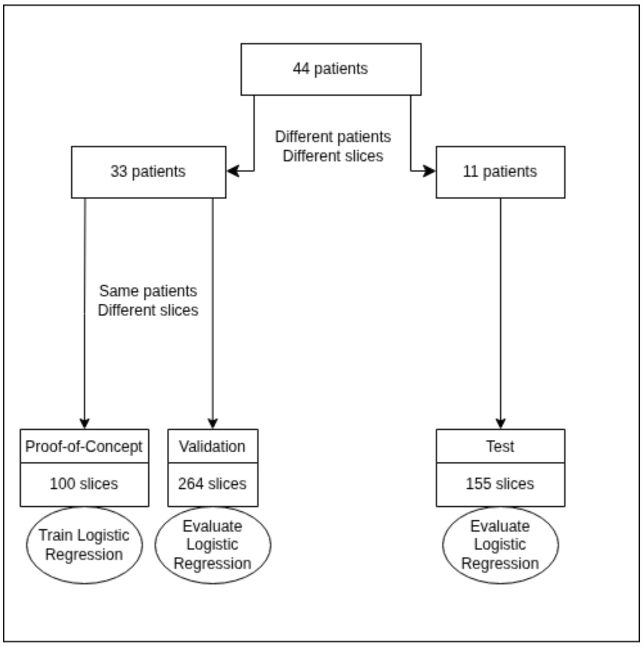

We thank the reviewer for this important and constructive comment. We acknowledge that the proof-of-concept (PoC) dataset excludes symmetrical lesions by design, while the independent test set contains a relatively high proportion of such cases (5 out of 11 patients), which contributes to the observed decrease in performance (AUC = 0.647). We agree that this highlights an important limitation of asymmetry-based approaches, as symmetrical lesions represent an inherent failure mode of the method.

The rationale behind this design was to first establish, in a controlled setting, whether asymmetry-based features carry discriminative information for lesion detection. The PoC dataset was therefore constructed to validate this hypothesis under favorable conditions. The inclusion of symmetrical lesions in the test set was not intentional but results from the limited cohort size and the constraint of maintaining strict patient-level independence between datasets and remains of the same order of magnitude as the overall prevalence in the cohort (~34%).

To clarify this point, we clarified the data partitioning strategy in the revised manuscript and now explicitly distinguish three datasets with complementary roles: (i) a PoC dataset used for feature selection and model training under controlled conditions, (ii) a validation dataset composed of independent images from the same patients to assess robustness, and (iii) an independent test dataset composed of unseen patients to evaluate generalization.

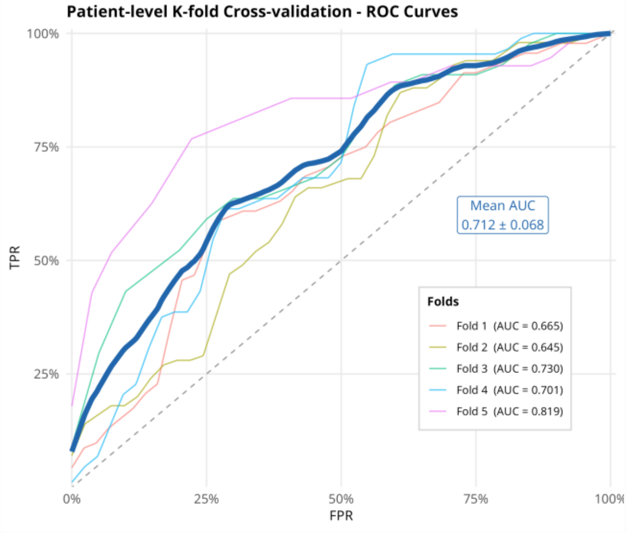

In addition, we performed a patient-level *k*-fold cross-validation on the combined PoC and validation datasets to assess whether the observed performance depends on this specific experimental design. The AUC values obtained across folds range from 0.645 to 0.819 (mean AUC = 0.712, standard deviation = 0.068). Furthermore, training a logistic regression model on the combined PoC and validation datasets and evaluating it on the independent test set yields an AUC of 0.665, consistent with the performance obtained when training on the PoC dataset alone.

Overall, these results suggest that performance remains broadly stable across different evaluation strategies, although variability across folds indicates sensitivity to sample composition. We have revised the Methods and Results sections to clarify these points and now explicitly discuss the impact of symmetrical lesions and dataset composition.

2. Author must provide the overall prevalence of symmetrical (e.g., horseshoe) lesions within the full 3TLAP cohort. Is 4/9 representative of the actual clinical distribution, or an artifact of an unfavorable random split? If it is an artifact, the test set should be resampled via cross- validation across the entire 44-patient cohort to provide a more accurate representation of generalized performance.

We thank the reviewer for this important request for clarification. After reviewing the full 3TLAP cohort (44 patients), we identified 15 patients with symmetrical lesions, corresponding to an overall prevalence of 34.1%. This proportion is consistent with the literature: Garg (2017) reports a prevalence of 36.1% of symmetrical lesions in a cohort of Crohn's disease patients with perianal involvement, supporting the clinical representativeness of our dataset.

Regarding the train/test split, the current test set (after adding two patients) includes 5 out of 11 patients with symmetrical lesions (45%). Although this proportion is higher than the overall prevalence (34.1%), it remains of the same order of magnitude and is consistent with variability expected from random sampling in a small cohort.

Given the limited number of patients, enforcing both strict patient-level independence and exact stratification with respect to lesion symmetry is challenging. To address the reviewer's concern, we complemented our

evaluation with a patient-level *k*-fold cross-validation performed on the combined proof-of-concept and validation datasets, which provides a more robust estimate of generalization performance across different patient subsets.

We have added the overall prevalence of symmetrical lesions in the Results section (test set evaluation) to allow direct comparison with the test set distribution and to better contextualize the reported performance.

3. The authors state that lesion-free control slices were taken from the perianal area of the same patients, outside the lesion range. In the discussion, you acknowledge this is a major limitation as nearby lesions may cause tissue displacement and low-level asymmetry. This is a critical methodological vulnerability. By definition, your controls are not truly healthy, and the baseline asymmetry distribution used to calculate the 3-Peak Score quantiles is inherently contaminated. The manuscript must explicitly justify why scans from genuinely healthy controls were not obtained (e.g., ethical constraints, retrospective cohort limitations). Furthermore, an analysis quantifying the distance between the control slices and the lesion slices should be added to see if low-level asymmetry decays the further the control slice is from the lesion range.

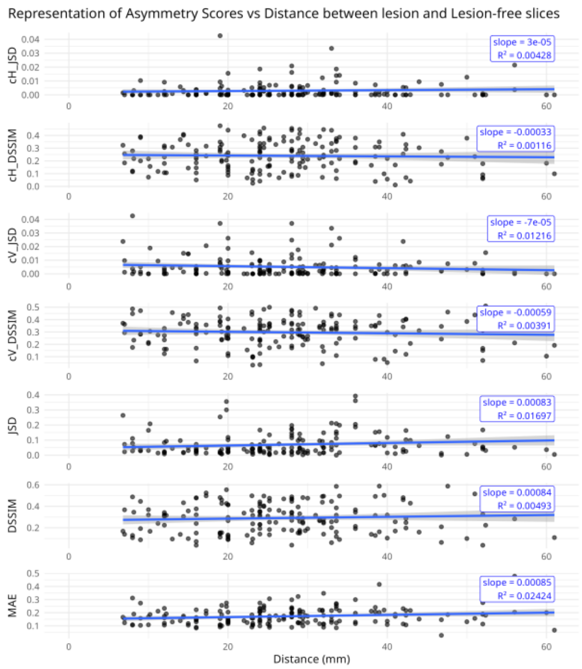

We agree that the absence of truly healthy control patients is a limitation inherent to this retrospective study. The 3TLAP cohort is composed exclusively of patients with perianal lesions related to Crohn's disease, and no MRI scans from healthy individuals were available. Obtaining such data would require a dedicated prospective acquisition or access to external datasets, as retrospective, disease-focused imaging cohorts typically do not include healthy control scans.

Despite this limitation, we provide additional analyses to assess the potential impact of using lesion-adjacent regions as controls. In particular, we quantified the distance between the center of the lesions and the control slices and examined whether asymmetry scores vary as a function of this distance. As shown in the corresponding figure, all evaluated asymmetry measures exhibit near-zero slopes and very low coefficients of determination ($R^2 < 0.025$), indicating no meaningful relationship between distance and asymmetry.

These results suggest that low-level asymmetry does not systematically decrease as control slices are located further from the lesion region within the studied range. We have added this analysis to the revised manuscript

and now explicitly discuss the potential contamination of control regions, as well as its implications for the interpretation of the asymmetry-based scores.

In addition, the use of lesion-adjacent regions as controls may introduce a conservative bias, as these regions can exhibit low-level asymmetry. As a result, the reference distribution used to define the 3-Peak Score thresholds may be shifted toward higher values, potentially reducing sensitivity to subtle asymmetries. Despite this, the model achieves good discrimination performance in the proof-of-concept setting (AUC = 0.858), suggesting that the asymmetry signal remains detectable even under these conditions.

These elements have been added to the revised manuscript and are now explicitly described and discussed in the Methods and Discussion sections.

4. The Data Availability statement mentions that data is available from the CHU of Rennes Institutional Data Access, listing Guillaume Bouguen as the contact. PLOS One policy typically requires a non-author institutional point of contact for data requests to ensure long-term access and prevent conflicts of interest. Guillaume Bouguen is listed as an author on this paper.

We added non-author contacts to our submission :

- Charlène Brochard (charlene.brochard@chu-rennes.fr)
- Amandine Landemaine (amandine.landemaine@chu-rennes.fr)

5. Please provide an institutional email address or an ethics committee contact that is independent of the authoring team for data requests.

The contact of the ethics committee will be added to the submission : cppse4@lyon.unicancer.fr

6. The abstract must explicitly state that the classification model struggles significantly with symmetrical lesions, as the AUC drop on the complete dataset is heavily tied to this limitation.

As suggested, we modified the abstract by adding precisions on the struggle of the model with symmetrical lesions and adding the drop of performance on test dataset:

"The classification model demonstrates promising diagnostic performance, achieving an Area Under Curve (AUC) greater than 0.85 on the proof-of-concept dataset. However, when making predictions on the validation and test dataset, the AUCs drop to 0.667 and 0.647 respectively. The model struggles to generalize to all kinds of lesions, particularly to classify lesions slices when symmetrical (horseshoe) lesions are observed."

7. In Section 2.3.2, the correlation formula is provided . Please ensure standard mathematical typesetting is utilized in the final manuscript submission and define exactly what pixel intensity ranges are used for Xi and Yi .

We thank the reviewer for this comment. The required modifications have been implemented in the revised manuscript, including standard mathematical typesetting of the correlation formula and clarification of the pixel intensity ranges used for *Xi* and *Yi*.

8. Figure 2: The adjusted p-values are listed using scientific notation (e.g., 3.6e-7). It is generally better formatting to use standard scientific notation (e.g., 3.6×10^{-7}) in publication-grade figures.

We applied the suggested modifications to Figure 2 in the revised version.

9. In the discussion, you mention an attempt to correlate asymmetry scores with clinical indices like the Van Assche score and MAGNIFI-CD, but note that too few medical reports included these values. You should briefly hypothesize how an asymmetry score might theoretically map to these indices (e.g., does a higher JSD correlate to more extensive tract branching?).

We thank the reviewer for this valuable and insightful suggestion. As suggested, we initially aimed to correlate asymmetry-based scores with clinical indices such as the Van Assche and MAGNIFI-CD scores; however, too few medical reports in our cohort included these annotations to enable a meaningful analysis.

From a theoretical perspective, asymmetry measures could reflect structural properties of the lesions, such as their spatial extent or complexity. For instance, higher asymmetry scores (e.g., based on JSD) might be associated with more extensive or irregular lesion patterns, potentially corresponding to higher clinical severity scores.

In this context, and in line with the reviewer's suggestion, one possible direction would be to compute asymmetry features across slices along the lesion range to reconstruct a three-dimensional profile of the lesion, which could then be compared to existing clinical indices.

We have added a paragraph in the Discussion section to outline these hypotheses and potential future directions.
