# Supplementary material for: Sequence-robust MRI asymmetry measures for the detection of anoperineal lesions in Crohn’s disease on axial pelvic MRI scans
Source: PLoS One. 2026 Jul 21;21(7):e0340243. doi: 10.1371/journal.pone.0340243 (PMC13387581; doi:10.1371/journal.pone.0340243)
Supplement: Peer review history

# Peer review: Decision Letter 0

**DOI:** 10.1371/journal.pone.0340243.r001

**Authors:** Khan Bahadar Khan

3 Mar 2026

PONE-D-25-64447

Sequence-independent MRI asymmetry measures for the detection of anoperineal lesions in Crohn’s disease on axial pelvic MRI scans.

PLOS One

Dear Dr. Le Cunff,

Thank you for submitting your manuscript to PLOS ONE. After careful consideration, we feel that it has merit but does not fully meet PLOS ONE’s publication criteria as it currently stands. Therefore, we invite you to submit a revised version of the manuscript that addresses the points raised during the review process.

Please submit your revised manuscript by Apr 17 2026 11:59PM. If you will need more time than this to complete your revisions, please reply to this message or contact the journal office at plosone@plos.org. When you're ready to submit your revision, log on to https://www.editorialmanager.com/pone/ and select the 'Submissions Needing Revision' folder to locate your manuscript file.

Please include the following items when submitting your revised manuscript:

- A letter that responds to each point raised by the academic editor and reviewer(s). You should upload this letter as a separate file labeled 'Response to Reviewers'.
- A marked-up copy of your manuscript that highlights changes made to the original version. You should upload this as a separate file labeled 'Revised Manuscript with Track Changes'.
- An unmarked version of your revised paper without tracked changes. You should upload this as a separate file labeled 'Manuscript'.

If you would like to make changes to your financial disclosure, please include your updated statement in your cover letter. Guidelines for resubmitting your figure files are available below the reviewer comments at the end of this letter.

If applicable, we recommend that you deposit your laboratory protocols in protocols.io to enhance the reproducibility of your results. Protocols.io assigns your protocol its own identifier (DOI) so that it can be cited independently in the future. For instructions see: https://journals.plos.org/plosone/s/submission-guidelines#loc-laboratory-protocols. Additionally, PLOS ONE offers an option for publishing peer-reviewed Lab Protocol articles, which describe protocols hosted on protocols.io. Read more information on sharing protocols at https://plos.org/protocols?utm_medium=editorial-email&utm_source=authorletters&utm_campaign=protocols.

We look forward to receiving your revised manuscript.

Kind regards,

Khan Bahadar Khan, Ph.D

Academic Editor

PLOS One

Journal Requirements:

When submitting your revision, we need you to address these additional requirements.

1. Please ensure that your manuscript meets PLOS ONE's style requirements, including those for file naming. The PLOS ONE style templates can be found at

https://journals.plos.org/plosone/s/file?id=wjVg/PLOSOne_formatting_sample_main_body.pdf and

https://journals.plos.org/plosone/s/file?id=ba62/PLOSOne_formatting_sample_title_authors_affiliations.pdf

2. Please update your submission to use the PLOS LaTeX template. The template and more information on our requirements for LaTeX submissions can be found at http://journals.plos.org/plosone/s/latex.

3. Thank you for stating the following in the Acknowledgments Section of your manuscript:

“We would also like to thank INRIA for supporting this work through doctoral funding”

We note that you have provided funding information that is not currently declared in your Funding Statement. However, funding information should not appear in the Acknowledgments section or other areas of your manuscript. We will only publish funding information present in the Funding Statement section of the online submission form.

Please remove any funding-related text from the manuscript and let us know how you would like to update your Funding Statement. Currently, your Funding Statement reads as follows:

“The author(s) received no specific funding for this work.”

Please include your amended statements within your cover letter; we will change the online submission form on your behalf.

4. Thank you for stating the following financial disclosure:

“We would also like to thank INRIA for supporting this work through doctoral funding”

At this time, please address the following queries:

a) Please clarify the sources of funding (financial or material support) for your study. List the grants or organizations that supported your study, including funding received from your institution.

b) State what role the funders took in the study. If the funders had no role in your study, please state: “The funders had no role in study design, data collection and analysis, decision to publish, or preparation of the manuscript.”

c) If any authors received a salary from any of your funders, please state which authors and which funders.

d) If you did not receive any funding for this study, please state: “The authors received no specific funding for this work.”

Please include your amended statements within your cover letter; we will change the online submission form on your behalf.

5. In the online submission form you indicate that your data is not available for proprietary reasons and have provided a contact point for accessing this data. Please note that your current contact point is a co-author on this manuscript. According to our Data Policy, the contact point must not be an author on the manuscript and must be an institutional contact, ideally not an individual. Please revise your data statement to a non-author institutional point of contact, such as a data access or ethics committee, and send this to us via return email. Please also include contact information for the third party organization, and please include the full citation of where the data can be found.

6. Please include your full ethics statement in the ‘Methods’ section of your manuscript file. In your statement, please include the full name of the IRB or ethics committee who approved or waived your study, as well as whether or not you obtained informed written or verbal consent. If consent was waived for your study, please include this information in your statement as well.

7. If the reviewer comments include a recommendation to cite specific previously published works, please review and evaluate these publications to determine whether they are relevant and should be cited. There is no requirement to cite these works unless the editor has indicated otherwise.

[Note: HTML markup is below. Please do not edit.]

Reviewers' comments:

Reviewer's Responses to Questions

**Comments to the Author**

1. Is the manuscript technically sound, and do the data support the conclusions?

The manuscript must describe a technically sound piece of scientific research with data that supports the conclusions. Experiments must have been conducted rigorously, with appropriate controls, replication, and sample sizes. The conclusions must be drawn appropriately based on the data presented.

Reviewer #1: No

Reviewer #2: Yes

Reviewer #3: No

**********

2. Has the statistical analysis been performed appropriately and rigorously?

Reviewer #1: No

Reviewer #2: No

Reviewer #3: No

**********

3. Have the authors made all data underlying the findings in their manuscript fully available?

The PLOS Data policy requires authors to make all data underlying the findings described in their manuscript fully available without restriction, with rare exception (please refer to the Data Availability Statement in the manuscript PDF file). The data should be provided as part of the manuscript or its supporting information, or deposited to a public repository. For example, in addition to summary statistics, the data points behind means, medians and variance measures should be available. If there are restrictions on publicly sharing data—e.g. participant privacy or use of data from a third party—those must be specified.

Reviewer #1: Yes

Reviewer #2: No

Reviewer #3: No

**********

4. Is the manuscript presented in an intelligible fashion and written in standard English?

PLOS ONE does not copyedit accepted manuscripts, so the language in submitted articles must be clear, correct, and unambiguous. Any typographical or grammatical errors should be corrected at revision, so please note any specific errors here.

Reviewer #1: Yes

Reviewer #2: Yes

Reviewer #3: Yes

**********

5. Review Comments to the Author

Please use the space provided to explain your answers to the questions above. You may also include additional comments for the author, including concerns about dual publication, research ethics, or publication ethics. (Please upload your review as an attachment if it exceeds 20,000 characters)

Reviewer #1: The study looks at an important clinical problem and the general idea makes sense. Using left–right asymmetry in MRI scans to detect lesions is logical, and the method is clearly explained. The early analyses show that some of the asymmetry measures are higher when lesions are present, and the localization results suggest that the approach could be useful in practice. However, the conclusions feel a bit stronger than what the data can fully support. The study only includes 44 patients, and the independent test group has just nine patients, which is quite small. This makes it hard to be confident that the method would work equally well in other hospitals or larger populations. Also, the Proof-of-Concept dataset was created using very clear and mostly asymmetric lesions, which likely made the method look better during development. When the model was applied to the full dataset and the test set, performance dropped, which shows that the real-world situation is more challenging. Overall, the results show that the idea is promising, but the claims about robustness, independence from MRI type, and clinical usefulness should be more cautious, since this is still an early-stage study with limited data.

The statistical methods used in the paper are reasonable for exploring the data, but they are not strong enough to fully support firm conclusions. The authors used appropriate tests like nonparametric comparisons, ANOVA, and PCA to look at patterns in the data, which is fine for exploratory work. However, the way the prediction model was evaluated is not fully convincing. It is not clearly shown whether proper cross-validation was done, especially at the patient level, raising concerns about optimistic bias and potential within-patient dependence among slices. Since multiple MRI slices come from the same patient, treating them as completely independent could make the results look better than they really are. The paper also does not report confidence intervals for key performance measures like AUC, precision, recall, or F1-score, so it is hard to judge how stable or uncertain the results are. The independent test set is also very small, which makes it difficult to draw strong conclusions about how well the model would perform in other settings. In addition, the authors interpret non-significant results about MRI type as proof that MRI type has no effect, but with such a small sample, it may simply be that the study does not have enough power to detect differences. Overall, the statistical approach needs stronger validation methods and clearer reporting to make the findings more reliable.

To enhance the statistical rigor of the manuscript, the authors should implement patient-level cross-validation. This approach would ensure that the model's performance is evaluated based on how well it generalizes to new patients, rather than just relying on the same data used for training. Additionally, the authors should provide bootstrapped confidence intervals for their performance metrics, such as AUC, precision, recall, and F1-score. To make the results more reliable, the authors should use stronger validation methods, such as checking performance at the patient level rather than at the slice level, and reporting confidence intervals. They should also clearly explain their assumptions about independence in the data. These steps would make the statistical conclusions more solid and trustworthy.

Reviewer #2: The manuscript tackles a clinically important and underexplored problem—automated assessment of anoperineal/perianal Crohn’s lesions on axial pelvic MRI—with an interpretable, anatomy-aligned asymmetry pipeline designed for small, heterogeneous cohorts. The workflow estimates a bilateral symmetry axis, applies Gaussian denoising and a Haar wavelet transform, then computes engineered left–right asymmetry measures for (i) patch classification via a logistic regression model and (ii) within-slice localization using a sliding window and a “3-Peak Score” (average of the top three normalized asymmetry scores) to identify the most likely lesion position along the y-axis. The emphasis on interpretability is a clear strength, and the inclusion of a localization task is a meaningful step toward workflow support rather than detection alone.

That said, parts of the current evaluation risk optimistic estimates and therefore weaken the strongest claims. In particular, the PoC dataset is used to select the “significant” asymmetry measures and to train the logistic regression model, and performance is also reported on that same PoC set. Please label this explicitly as resubstitution/proof-of-concept performance and complement it with patient-level cross-validation (ideally nested if feature selection and normalization steps are repeated) or a proper internal validation split, while keeping the held-out test patients fully untouched.

Relatedly, please address the dependence structure: the dataset contains multiple sequences and slices per patient, yet key metrics are presented at the patch level as if observations were independent. Even with the “patient effect” analysis in PCA space, the classification and localization results would be more convincing with patient-level reporting and cluster-aware uncertainty.

The clinical framing and interpretation of test-set results also need strengthening. The held-out test classification performance is modest, so the manuscript would benefit from a sharper, concrete use case (triage, reader aid, or quality control) and at least simple baselines beyond the proposed asymmetry features. In addition, the test split contains a high proportion of symmetric (“horseshoe”) lesions (4/9 patients), which is described as an “unfavorable random split” and used to explain degraded classification performance. Because symmetry is an acknowledged failure mode for asymmetry-based detection, please frame this explicitly as a key limitation, and describe the splitting strategy clearly (and ideally use stratified or repeated patient-level splitting).

The claim of MRI-type independence needs more direct support. The paper argues that scores are “not sensitive” to MRI type largely via PCA and (Welch’s) ANOVA on PCA dimensions, and then makes broader statements about sequence independence. If MRI-type independence is a central claim, please support it with stratified performance (or at least subgroup summaries) by sequence type, rather than relying on PCA/ANOVA alone.

Please also improve reproducibility and method specificity. Patch sizes are given in centimeters (1 cm × 7 cm) and the localization window steps by one pixel, but the cm-to-pixel conversion across varying DICOM spacings is not fully specified; please add exact implementation details. The beta-biased null for localization is described as a beta-like distribution fit to PoC lesion positions; please report how parameters are estimated and ensure this prior is fit only on training data. Finally, please consider sharing code or derived features (even if raw data cannot be public due to privacy restrictions) to improve reproducibility.

Reviewer #3: The authors present a computational method to detect and localize anoperineal lesions in Crohn's disease using axial pelvic MRI scans. Addressing the limitations of deep learning on small, heterogeneous medical datasets, the authors utilize a simpler, interpretable approach based on asymmetry measures (JSD, MAE, DSSIM) combined with Gaussian filtering and wavelet transforms. Evaluated on a cohort of 44 patients (134 MRI sequences), the logistic regression classifier achieved an AUC of 0.858 on a curated Proof-of-Concept (PoC) dataset, but dropped to an AUC of 0.594 on a test set containing symmetrical lesions. However, the localization task showed promise, identifying the lesion within roughly 1 to 1.2 cm of its actual center across datasets

**********

6. PLOS authors have the option to publish the peer review history of their article (what does this mean?). If published, this will include your full peer review and any attached files.

If you choose “no”, your identity will remain anonymous but your review may still be made public.

**Do you want your identity to be public for this peer review?**  For information about this choice, including consent withdrawal, please see our Privacy Policy.

Reviewer #1: No

Reviewer #2: No

Reviewer #3: No

**********

[NOTE: If reviewer comments were submitted as an attachment file, they will be attached to this email and accessible via the submission site. Please log into your account, locate the manuscript record, and check for the action link "View Attachments". If this link does not appear, there are no attachment files.]

To ensure your figures meet our technical requirements, please review our figure guidelines: https://journals.plos.org/plosone/s/figures

You may also use PLOS’s free figure tool, NAAS, to help you prepare publication quality figures: https://journals.plos.org/plosone/s/figures#loc-tools-for-figure-preparation.

NAAS will assess whether your figures meet our technical requirements by comparing each figure against our figure specifications.

Attachment Submitted filename: Recommendation.pdf

# Author comment: Author response to Decision Letter 1

**DOI:** 10.1371/journal.pone.0340243.r002

20 Apr 2026

We would like to thank the reviewers and the editor for their comments on the manuscript, which have helped us improve it significantly. Our responses to the reviewers are provided in the file Response_to_reviewers.pdf and have been incorporated into the revised manuscript.

Attachment Submitted filename: Response_to_reviewers.pdf

# Peer review: Decision Letter 1

**DOI:** 10.1371/journal.pone.0340243.r003

**Authors:** Khan Bahadar Khan, Khan Bahadar Khan

18 Jun 2026

Sequence-robust MRI asymmetry measures for the detection of anoperineal lesions in Crohn’s disease on axial pelvic MRI scans.

PONE-D-25-64447R1

Dear Dr. Le Cunff,

We’re pleased to inform you that your manuscript has been judged scientifically suitable for publication and will be formally accepted for publication once it meets all outstanding technical requirements.

Within one week, you’ll receive an e-mail detailing the required amendments. When these have been addressed, you’ll receive a formal acceptance letter and your manuscript will be scheduled for publication.

An invoice will be generated when your article is formally accepted. Please note, if your institution has a publishing partnership with PLOS and your article meets the relevant criteria, all or part of your publication costs will be covered. Please make sure your user information is up-to-date by logging into Editorial Manager at Editorial Manager® and clicking the ‘Update My Information' link at the top of the page. For questions related to billing, please contact billing support.

If your institution or institutions have a press office, please notify them about your upcoming paper to help maximize its impact. If they’ll be preparing press materials, please inform our press team as soon as possible -- no later than 48 hours after receiving the formal acceptance. Your manuscript will remain under strict press embargo until 2 pm Eastern Time on the date of publication. For more information, please contact onepress@plos.org.

Kind regards,

Khan Bahadar Khan, Ph.D

Academic Editor

PLOS One

Additional Editor Comments (optional):

Reviewers' comments:

Reviewer's Responses to Questions

**Comments to the Author**

1. If the authors have adequately addressed your comments raised in a previous round of review and you feel that this manuscript is now acceptable for publication, you may indicate that here to bypass the “Comments to the Author” section, enter your conflict of interest statement in the “Confidential to Editor” section, and submit your "Accept" recommendation.

Reviewer #1: All comments have been addressed

Reviewer #2: (No Response)

Reviewer #3: All comments have been addressed

**********

2. Is the manuscript technically sound, and do the data support the conclusions?

The manuscript must describe a technically sound piece of scientific research with data that supports the conclusions. Experiments must have been conducted rigorously, with appropriate controls, replication, and sample sizes. The conclusions must be drawn appropriately based on the data presented.

Reviewer #1: Yes

Reviewer #2: (No Response)

Reviewer #3: Yes

**********

3. Has the statistical analysis been performed appropriately and rigorously?

Reviewer #1: Yes

Reviewer #2: (No Response)

Reviewer #3: Yes

**********

4. Have the authors made all data underlying the findings in their manuscript fully available?

The PLOS Data policy requires authors to make all data underlying the findings described in their manuscript fully available without restriction, with rare exception (please refer to the Data Availability Statement in the manuscript PDF file). The data should be provided as part of the manuscript or its supporting information, or deposited to a public repository. For example, in addition to summary statistics, the data points behind means, medians and variance measures should be available. If there are restrictions on publicly sharing data—e.g. participant privacy or use of data from a third party—those must be specified.

Reviewer #1: Yes

Reviewer #2: (No Response)

Reviewer #3: Yes

**********

5. Is the manuscript presented in an intelligible fashion and written in standard English?

PLOS ONE does not copyedit accepted manuscripts, so the language in submitted articles must be clear, correct, and unambiguous. Any typographical or grammatical errors should be corrected at revision, so please note any specific errors here.

Reviewer #1: Yes

Reviewer #2: (No Response)

Reviewer #3: Yes

**********

6. Review Comments to the Author

Please use the space provided to explain your answers to the questions above. You may also include additional comments for the author, including concerns about dual publication, research ethics, or publication ethics. (Please upload your review as an attachment if it exceeds 20,000 characters)

Reviewer #1: The authors have adequately addressed the major concerns raised in the previous review. The revised manuscript is significantly improved, particularly with the addition of patient-level validation analyses, leave-one-patient-out evaluation, and clearer discussion of the limitations related to small sample size and symmetric lesions. The statistical methodology is now more transparent, and the conclusions are presented in a more balanced and appropriate manner.

I also appreciate that the authors clarified the distinction between the Proof-of-Concept, validation, and test datasets, and softened some of the stronger claims regarding MRI-sequence independence and generalizability. The additional discussion regarding false negatives, especially in symmetric lesions, strengthens the interpretation of the results.

Although limitations remain, including the relatively small cohort size and absence of confidence intervals for some performance metrics, these issues are now appropriately acknowledged and do not substantially undermine the value of the study as a proof-of-concept investigation.

Overall, I believe the manuscript is suitable for publication in its revised form.

Reviewer #2: I thank the authors for their careful and detailed responses to the previous reviews. The revised manuscript is substantially improved, and I appreciate the constructive efforts to address the reviewers’ concerns.

Reviewer #3: This paper has been revised and all comments are addressed well. I have no additonal comments and i recoomend this paper for acceptance.

**********

7. PLOS authors have the option to publish the peer review history of their article (what does this mean?). If published, this will include your full peer review and any attached files.

If you choose “no”, your identity will remain anonymous but your review may still be made public.

**Do you want your identity to be public for this peer review?**  For information about this choice, including consent withdrawal, please see our Privacy Policy.

Reviewer #1: No

Reviewer #2: No

Reviewer #3: **Yes:** Sanaullah Sajid

**********

# Editor report: Acceptance letter

**DOI:** 10.1371/journal.pone.0340243.r004

**Authors:** Khan Bahadar Khan, Khan Bahadar Khan

PONE-D-25-64447R1

PLOS One

Dear Dr. Le Cunff,

I'm pleased to inform you that your manuscript has been deemed suitable for publication in PLOS One. Congratulations! Your manuscript is now being handed over to our production team.

At this stage, our production department will prepare your paper for publication. This includes ensuring the following:

* All references, tables, and figures are properly cited

* All relevant supporting information is included in the manuscript submission,

* There are no issues that prevent the paper from being properly typeset

You will receive further instructions from the production team, including instructions on how to review your proof when it is ready. Please keep in mind that we are working through a large volume of accepted articles, so please give us a few days to review your paper and let you know the next and final steps.

Lastly, if your institution or institutions have a press office, please let them know about your upcoming paper now to help maximize its impact. If they'll be preparing press materials, please inform our press team within the next 48 hours. Your manuscript will remain under strict press embargo until 2 pm Eastern Time on the date of publication. For more information, please contact onepress@plos.org.

You will receive an invoice from PLOS for your publication fee after your manuscript has reached the completed accept phase. If you receive an email requesting payment before acceptance or for any other service, this may be a phishing scheme. Learn how to identify phishing emails and protect your accounts at https://explore.plos.org/phishing.

If we can help with anything else, please email us at customercare@plos.org.

Thank you for submitting your work to PLOS ONE and supporting open access.

Kind regards,

PLOS ONE Editorial Office Staff

on behalf of

Dr. Khan Bahadar Khan

Academic Editor

PLOS One
